# High Persister Mutants in *Mycobacterium tuberculosis*

**DOI:** 10.1371/journal.pone.0155127

**Published:** 2016-05-13

**Authors:** Heather L. Torrey, Iris Keren, Laura E. Via, Jong Seok Lee, Kim Lewis

**Affiliations:** 1 Department of Biology, Northeastern University, Boston, Massachusetts, United States of America; 2 Tuberculosis Research Section, Laboratory of Clinical Infectious Diseases, National Institute of Allergy and Infectious Diseases, National Institutes of Health, Bethesda, Maryland, United States of America; 3 International Tuberculosis Research Center, Changwon, Republic of Korea; The Scripps Research Institute and Sorrento Therapeutics, Inc., UNITED STATES

## Abstract

*Mycobacterium tuberculosis* forms drug-tolerant persister cells that are the probable cause of its recalcitrance to antibiotic therapy. While genetically identical to the rest of the population, persisters are dormant, which protects them from killing by bactericidal antibiotics. The mechanism of persister formation in *M*. *tuberculosis* is not well understood. In this study, we selected for high persister (*hip*) mutants and characterized them by whole genome sequencing and transcriptome analysis. In parallel, we identified and characterized clinical isolates that naturally produce high levels of persisters. We compared the *hip* mutants obtained *in vitro* with clinical isolates to identify candidate persister genes. Genes involved in lipid biosynthesis, carbon metabolism, toxin-antitoxin systems, and transcriptional regulators were among those identified. We also found that clinical *hip* isolates exhibited greater *ex vivo* survival than the low persister isolates. Our data suggest that *M*. *tuberculosis* persister formation involves multiple pathways, and *hip* mutants may contribute to the recalcitrance of the infection.

## Introduction

Over one third of the global human population is infected with *Mycobacterium tuberculosis*, the etiological agent of tuberculosis [1]. While up to 90% of tuberculosis infections are latent, with no clinical symptoms, they remain a concern due to their potential for reactivation [2], [3]. Treatment of active tuberculosis requires at least six months of antibiotic therapy. This lengthy treatment as well as the vast reservoir of latent infection is likely due to the presence of persister cells [4].

Persisters form a subpopulation of phenotypically drug tolerant cells. Unlike resistant mutants, persisters do not grow in the presence of antibiotics. Upon regrowth, persisters reestablish a population that retains the same susceptibility as the original population [5]. Persisters are produced by all bacterial species studied to date, but much of what we know about them is based on studies of *Escherichia coli*. Persisters are non-growing cells [6] whose gene expression profile [7] and low levels of translation [8] indicate that they are in a dormant state. The target pathways of antibiotics are inactive in dormant cells, which accounts for their tolerance to drug exposure [8]. In *E*. *coli*, pathways leading to dormancy are highly redundant and largely depend on the action of toxin-antitoxin (TA) modules [7], [9]. For example, protein synthesis inhibition by the HipA toxin [10], [11], a kinase [12] and by at least 10 different mRNA endonucleases such as RelE, MazF and YafQ [7], [9], [13], [14] leads to dormancy. In addition, damage of DNA induces the SOS response and expression of the TisB toxin [15], an endogenous antimicrobial peptide [16] that causes persister formation by opening an ion channel that decreases the proton motive force and ATP levels leading to a dormant, drug-tolerant state [17]. Typically overexpression of a persister gene leads to shutdown of an important cellular function, which results in drug tolerant persisters. Apart from TA modules, additional genes have been implicated in persister formation in *E*. *coli* including those involved in glycerol and nucleotide metabolism as well as some global regulators [18], [19], [20].

Transcriptome analysis of *M*. *tuberculosis* persister cells highlighted a metabolic downshift and upregulation of TA modules that is consistent with that observed in *E*. *coli* [7], [21]. In *M*. *tuberculosis*, there are close to 80 TA modules identified so far [22], [23], indicating a potential for an extremely high level of redundancy in mechanisms governing dormancy. Direct evidence of TA systems playing a role in drug tolerance of *M*. *tuberculosis* has been reported. Ectopic overexpression of three individual *E*. *coli relE* homologues (Rv1246c, Rv2866, Rv3358) was found to increase drug tolerance [24]. Interestingly, *relE* overexpression effects are drug and isoform specific, which suggests that more than one subpopulation of persisters exist. Other mechanisms responsible for differential antibiotic susceptibility of mycobacteria include asymmetrical growth during cell division [25], stochastic expression of genes affecting antimicrobial action such as catalase-peroxidase *katG*, which activates the prodrug isoniazid and affects drug tolerance in a subset of cells [26], and changes that affect storage lipid accumulation [27] such as shifts in carbon flux through the TCA cycle [28]. The latter points to alterations in metabolism as a key factor influencing drug tolerance in *M*. *tuberculosis*.

While a persister cell is in a temporary state, the proportion of persisters within a population is relatively stable. Typically, the frequency of persister formation increases with cell density and reaches about 1% in stationary phase [5], [21], [29]. The frequency of persister formation can also change with mutations. For example in *E*. *coli*, a gain of function mutation in the HipA toxin, obtained through *in vitro* mutagenesis, increases the level of persister production 1,000-fold [30]. In *Salmonella typhimurium*, a high persister (*hip*) mutation in the RelB toxin also increases persister production over 1,000-fold [31]. Selection for *hip* mutants can also occur in a clinical setting. Antibiotic treatment has been shown to select for *hip* mutants in patients with *Candida albicans* biofilms [32] or with *Pseudomonas aeruginosa* infection [33]. In *E*. *coli*, gain of function HipA mutants were recently discovered in clinical isolates and shown to have increased levels of persisters in an *ex vivo* model of infection [34]. These findings link persisters to clinical manifestation of disease. To date no *hip* mutants of *M*. *tuberculosis* have been reported. Here we report identification of *hip* mutants of *M*. *tuberculosis* obtained from an *in vitro* selection, and from a screen of clinical isolates.

## Results

### Characterization of *hip* mutants obtained *in vitro*

#### *Hip* phenotype selection

**Fig 1:**
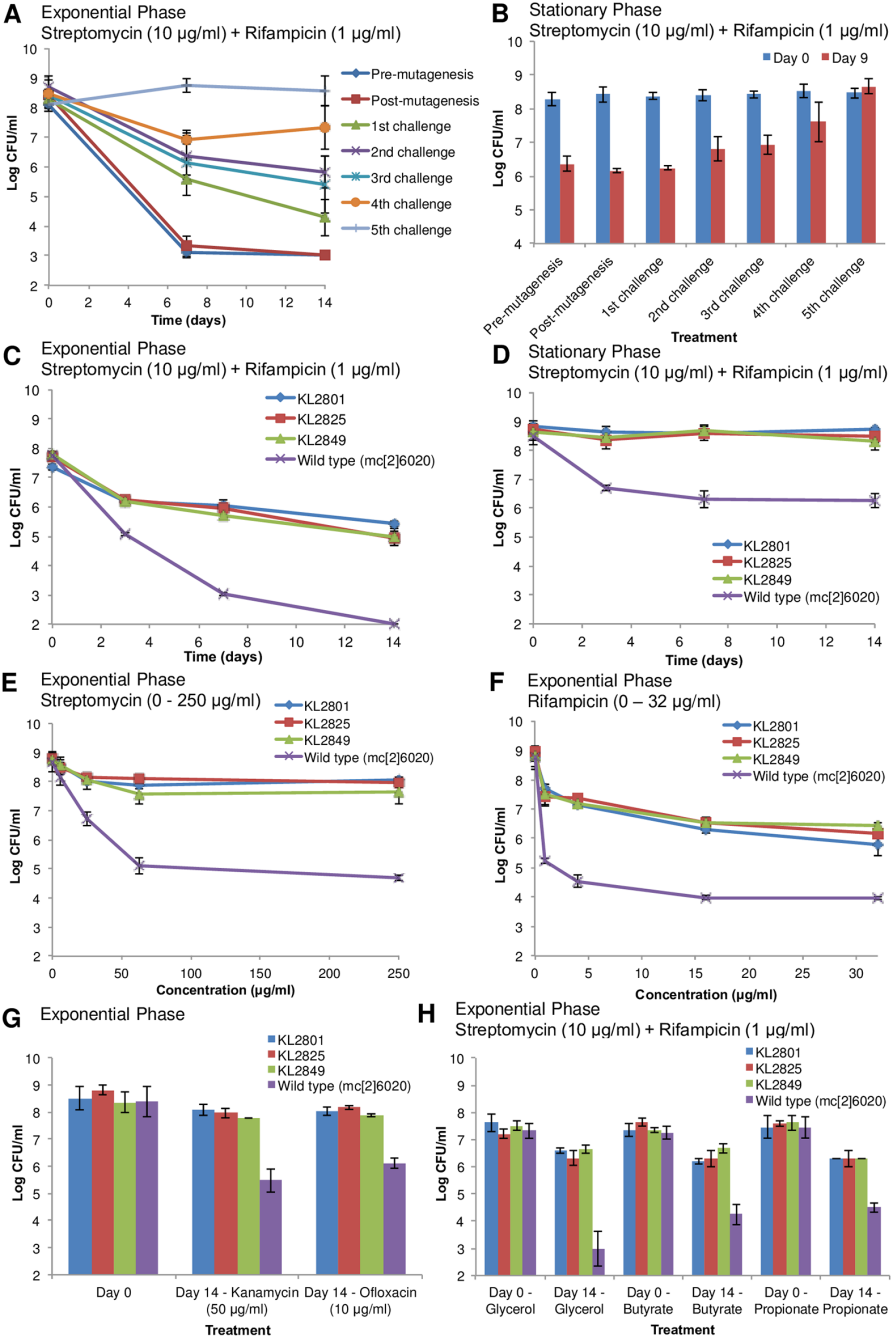
Characterization of *hip* mutants obtained *in vitro*. Persister assays, performed by antibiotic treatment with streptomycin (10 μg/ml) and rifampicin (1 μg/ml) for 14 days, reveal the number of drug tolerant persister cells based on CFU counts. Exponential (A) and stationary phase (B) treatment of mutagenized strain mc^2^6020 at each stage of the *hip* mutant selection process. Time-dependent persister assays in exponential (C) and stationary phase (D) with independent mutants KL2801, KL2825, KL2849, and wild type strain (mc^2^6020). Late exponential phase cultures were treated with various concentrations of streptomycin (E) or rifampicin (F) or with antibiotics not used in the selection process, kanamycin (50 μg/ml) or ofloxacin (10 μg/ml) (G). Cultures grown in minimal media with glycerol, butyrate, or propionate as the sole carbon source were treated in exponential phase (H). Data represent the average of three biological replicates and the error bars represent standard deviation.

To identify genes responsible for persister formation, we mutagenized *M*. *tuberculosis* mc^2^6020, an auxotroph of H37Rv [35], and selected for mutants surviving treatment with a lethal dose of streptomycin and rifampicin. With each round of selection, the level of persisters surviving antibiotic treatment increased in both exponential and stationary phase (Fig 1A and 1B). Three representative mutant strains were characterized further. The strains produced 100- to 1,000-fold more persisters than the parental wild type strain (mc^2^6020) in time-dependent (Fig 1C and 1D) and concentration-dependent antibiotic treatment (Fig 1E and 1F). In addition, the mutants consistently produced significantly more (*p*-value < 0.05) persisters than the wild type (mc^2^6020) when treated with antibiotics belonging to different classes that were not used in the selection process (Fig 1G), and when grown on various carbon sources (glycerol, butyrate, and propionate) (Fig 1H). For each mutant, neither minimum inhibitory concentration (MIC) (S4 Table) nor growth rate (S5 Table) differed significantly from the wild type (mc^2^6020) (*p*-value < 0.05). These selection criteria ruled out the possibility that increased drug resistance or growth defects were responsible for increased survival. Taken together, these results show that the *hip* mutants selected *in vitro* are multidrug tolerant and that their phenotype is independent of antibiotic class, antibiotic concentration, growth phase, or carbon source.

#### Whole genome sequencing and transcriptome analysis to identify candidate genes

**Fig 2:**
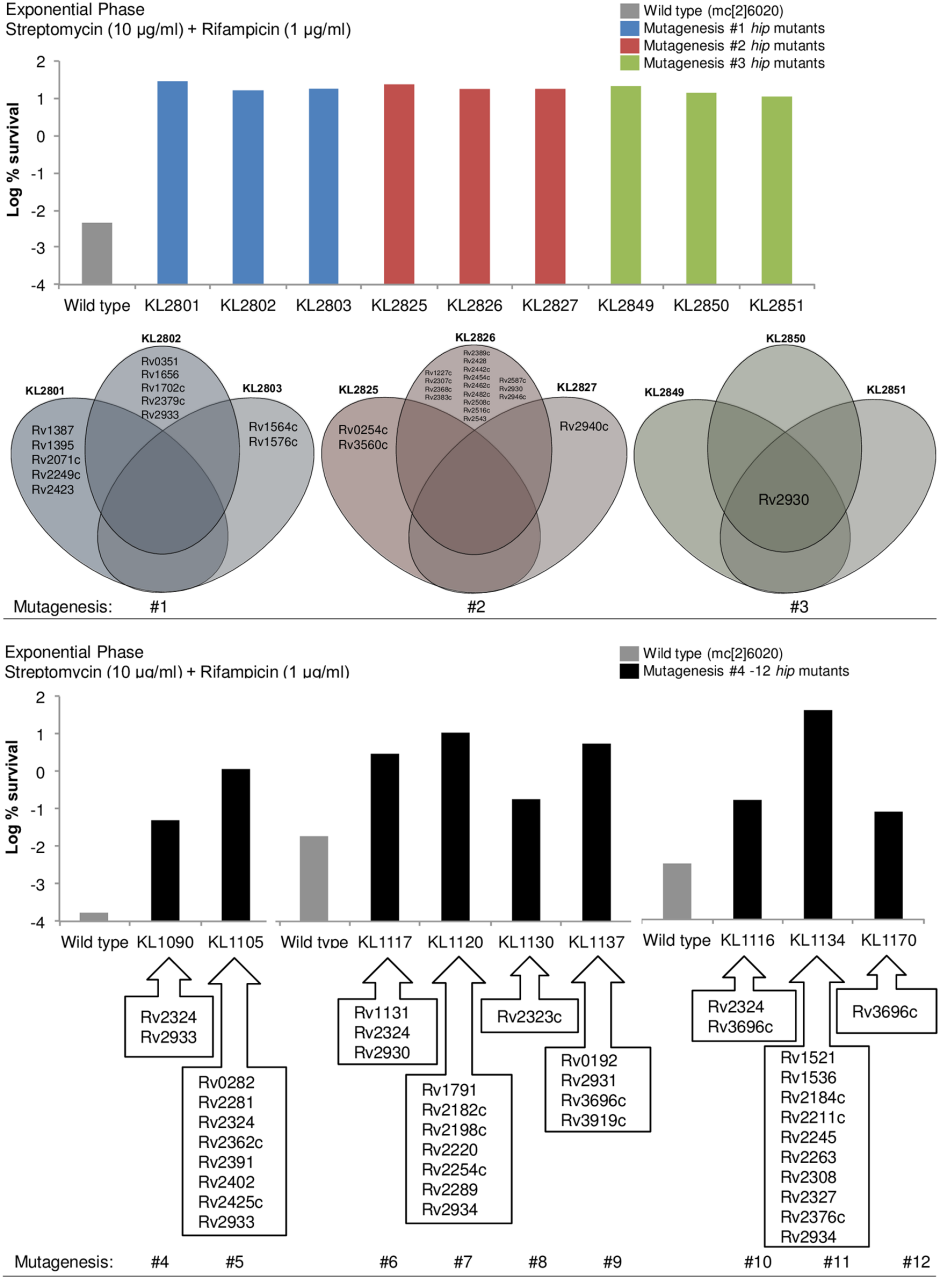
Genetic analysis of *hip* mutants obtained *in vitro*. Representative antibiotic survival plots of 18 *hip* mutant strains, obtained from 12 independent mutageneses, are presented along with lists of genes containing non-synonymous mutations within each strain.

The genomes of 18 *hip* mutants derived from 12 independent mutageneses were sequenced by Illumina technology and found to carry between one and 16 non-synonymous mutations (Fig 2 and Table 1). In one case, three mutants derived from the same mutagenesis (KL2849, KL2850, KL2851) contained identical non-synonymous mutations while strains derived from independent mutageneses contained mostly unique mutations. Since gene expression information could help elucidate the potential involvement of a candidate gene in the *hip* phenotype, transcriptome analysis of stationary phase *hip* mutants was carried out (S6 Table). Interestingly, transcriptome analysis showed high similarity in gene expression in the three *hip* mutants (KL2801, KL2825, KL2849) (S7 Table) even though there was no overlap in non-synonymous mutations (Table 1). This suggested that they each contained mutations in a common pathway. In KL2849, the single non-synonymous mutation in fatty-acid-CoA ligase *fadD26* (G74*) was an interesting candidate. *FadD26* is involved in biosynthesis of phthiocerol dimycocerosate (PDIM), an important virulence lipid. Subsequent total lipid analysis confirmed an absence of PDIM among the three strains (S1 Fig). With KL2801 and KL2825, while there was no mutation in the ORFs of its biosynthetic pathway, it is possible that a mutation in a promoter or regulatory region is responsible for the PDIM-null phenotype. Sequence analysis revealed multiple mutations and single non-synonymous mutations in genes associated with PDIM biosynthesis in several other *hip* mutants (Table 1).

**Table 1:** Non-synonymous mutations identified in individual *in vitro hip* mutant strains by whole genome sequencing.

Strain	Gene	Annotation	Location	Reference	Mutant	Change
KL2801	Rv1387	PPE family protein	1563341	C	T	P525S
	Rv1395	transcriptional regulator	1571669	C	T	P208L
	Rv2071c	precorrin-4 c11-methyltransferase cobM	2328463	C	T	A172V
	Rv2249c	glycerol-3-phosphate dehydrogenase glpD1	2523381	C	T	R471W
	Rv2423	hypothetical protein	2719708	G	A	V38M
KL2802	Rv0351	chaperone grpE	422212	G	A	M168I
	Rv1656	ornithine carbamoyltransferase argF	1870565	C	T	A215V
	Rv1702c	conserved hypothetical protein	1927776	G	A	G267D
	Rv2379c	peptide synthetase mbtF	2661260	T	C	F276L
	Rv2933	phenolpthiocerol synthesis type-I polyketide synthase ppsC	3261616	T	C	W1978R
KL2803	Rv1564c	maltooligosyltrehalose synthase treX	1769662	C	T	A647V
	Rv1576c	phiRv1 phage protein	1781577	C	T	T163I
KL2825	Rv0254c	bifunctional cobalamin biosynthesis protein cobU	305878	G	A	A158T
	Rv3560c	acyl-CoA dehydrogenase fadE30	4000449	C	T	R381W
KL2826	Rv1227c	transmembrane protein	1370665	G	A	W54*
	Rv2307c	conserved hypothetical protein	2578588	C	T	P37S
	Rv2368c	phosphate starvation-inducible protein phoH1	2649391	C	T	T195M
	Rv2383c	phenyloxazoline synthase mbtB	2671835	C	T	P1335S
	Rv2389c	resuscitation-promoting factor rpfD	2683423	C	T	A97V
	Rv2428	alkyl hydroperoxide reductase C protein ahpC	2726541	G	A	E117K
	Rv2442c	50S ribosomal protein L21 rplU	2740088	C	T	R92C
	Rv2454c	oxidoreductase beta subunit	2754530	C	T	P73S
	Rv2462c	trigger factor protein tig	2764088	C	T	P402S
	Rv2482c	glycerol-3-phosphate acyltransferase plsB2	2788623	C	T	L221F
	Rv2508c	conserved alanine and leucine rich membrane protein	2824394	C	T	A67V
	Rv2516c	hypothetical protein	2832738	C	T	A259V
	Rv2543	lipoprotein lppA	2866837	G	A	V124I
	Rv2587c	protein-export membrane protein secD	2915001	C	T	P246S
	Rv2930	fatty-acid-CoA ligase fadD26	3243916	G	T	G74*
	Rv2946c	polyketide synthase pks1	3293047	G	A	E1103K
KL2827	Rv2940c	multifunctional mycocerosic acid synthase membrane-associated mas	3280534	T	C	S728P
KL2849	Rv2930	fatty-acid-CoA ligase fadD26	3243916	G	T	G74*
KL2850	Rv2930	fatty-acid-CoA ligase fadD26	3243916	G	T	G74*
KL2851	Rv2930	fatty-acid-CoA ligase fadD26	3243916	G	T	G74*
KL1090	Rv2324	transcriptional regulator, asnC-family	2596356	A	G	D8G
	Rv2933	phenolpthiocerol synthesis type-I polyketide synthase ppsC	3257052	AC	AGACGAATGC…	
KL1105	Rv0282	conserved hypothetical protein	342139	G	T	V4L
	Rv2281	phosphate-transport permease pitB	2554762	G	A	W530*
	Rv2324	transcriptional regulator, asnC-family	2596559	G	A	G76S
	Rv2362c	conserved hypothetical protein	2643744	C	T	T172I
	Rv2391	ferredoxin-dependent nitrite reductase nirA	2685163	G	A	G162E
	Rv2402	conserved hypothetical protein	2699244	G	A	W239*
	Rv2425c	conserved hypothetical protein	2722000	C	T	L437F
	Rv2933	phenolpthiocerol synthesis type-I polyketide synthase ppsC	3257052	AC	AGACGAATGC…	
KL1116	Rv2324	transcriptional regulator, asnC-family	2596492	C	A	F52L
	Rv3696c	glycerol kinase glpK	4139183	A	AC	
KL1117	Rv1131	citrate synthase I gltA1	1257138	G	A	G335E
	Rv2324	transcriptional regulator, asnC-family	2596619	G	T	A95S
	Rv2930	fatty-acid-CoA ligase fadD26	3244737	CA	C	
KL1120	Rv1791	PE family protein	2030036	G	A	D44N
	Rv2182c	1-acylglycerol-3-phosphate o-acyltransferase	2445124	G	A	G68D
	Rv2198c	membrane protein mmpS3	2462848	G	A	G66D
	Rv2220	glutamine synthetase glnA1	2487928	C	T	S104F
	Rv2254c	membrane protein	2528774	G	A	A67T
	Rv2289	cdp-diacylglycerol pyrophosphatase cdh	2562042	C	T	A122V
	Rv2934	phenolpthiocerol synthesis type-I polyketide synthase ppsD	3266422	T	C	L1391P
KL1130	Rv2323c	conserved hypothetical protein	2596217	G	A	R17H
KL1134	Rv1521	fatty-acid-CoA ligase fadD25	1713505	G	A	G401R
	Rv1536	isoleucyl-tRNA synthetase ileS	1738703	G	A	E728K
	Rv2184c	conserved hypothetical protein	2446234	C	T	A237V
	Rv2211c	aminomethyltransferase gcvT	2476797	C	T	P128S
	Rv2245	3-oxoacyl-[acyl-carrier protein] synthase 1 kasA	2519289	G	A	G391D
	Rv2263	oxidoreductase	2535777	G	A	R45H
	Rv2308	conserved hypothetical protein	2580606	G	A	G62D
	Rv2327	conserved hypothetical protein	2600309	G	A	E107K
	Rv2376c	low molecular weigKL protein antigen cfp2	2655937	C	T	A59V
	Rv2934	phenolpthiocerol synthesis type-I polyketide synthase ppsD	3264484	C	A	A745E
KL1137	Rv0192	conserved hypothetical protein	223958	C	T	P131L
	Rv2931	phenolpthiocerol synthesis type-I polyketide synthase ppsA	3247786	G	A	G780D
	Rv3696c	glycerol kinase glpK	4138776	G	A	G326D
	Rv3919c	glucose-inhibited division protein B gid	4407977	G	A	G75S
KL1170	Rv3696c	glycerol kinase glpK	4138299	G	A	R485Q

**Fig 3:**
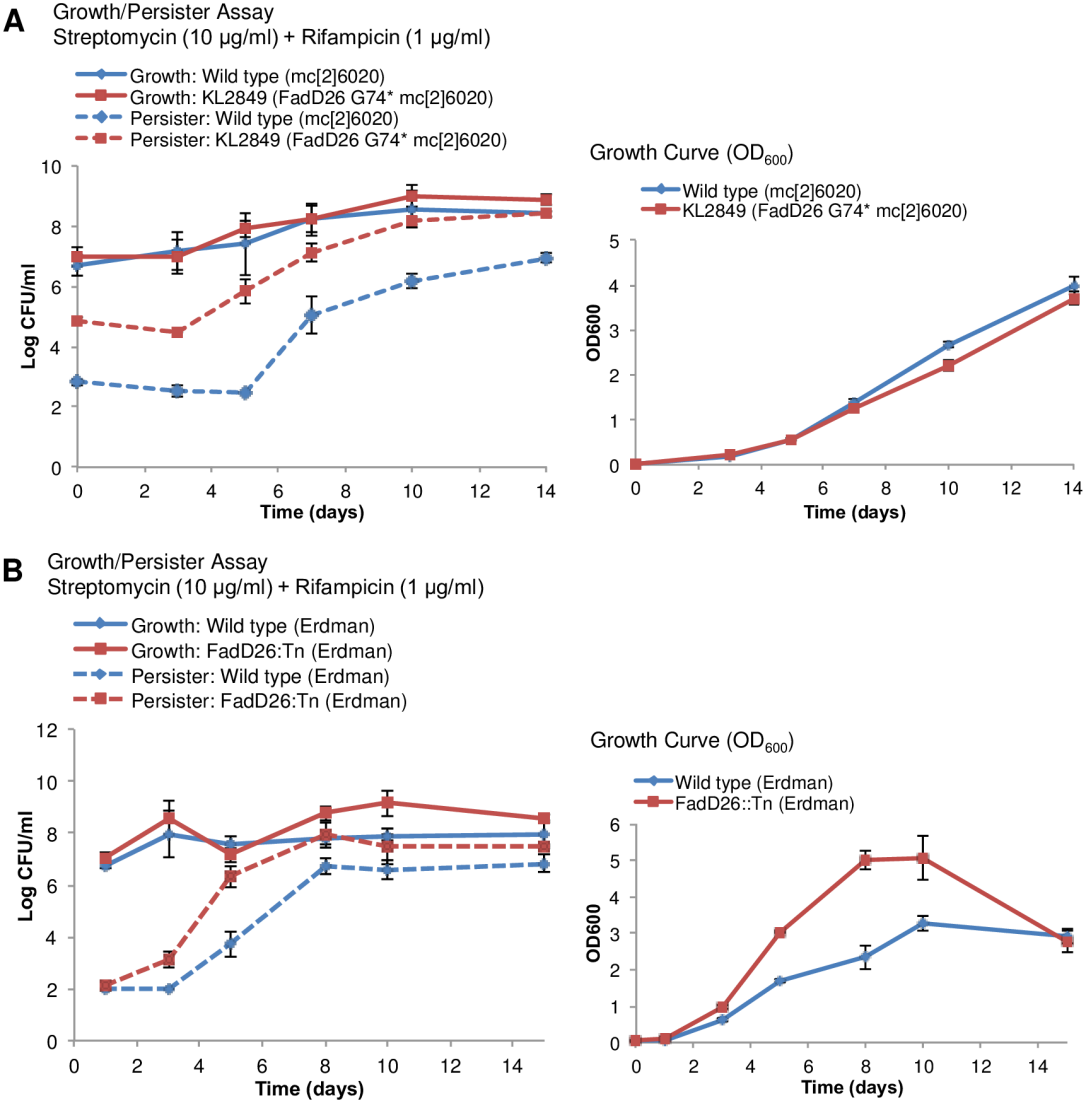
Growth and persister assays of *fadD26* PDIM mutant strains. PDIM mutant strain KL2849 (*fadD26* G74*) and wild type (mc^2^6020) (A) or *fadD26*::Tn and wild type (Erdman) (B) were grown (solid lines) and treated with streptomycin (10 μg/ml) and rifampicin (1 μg/ml) at the indicated time points for 14 days (dashed lines). Bacterial survival was determined by plating for CFU. Growth curves were also monitored based on OD_600_ readings. The values are an average of three biological replicates and error bars represent standard deviation.

To test whether the loss of PDIM is associated with the *hip* phenotype, we compared the growth-persister pattern of the *fadD26* (G74*) PDIM-null *hip* mutant (KL2849) and the parental wild type strain (mc^2^6020) with that of a previously published *fadD26* transposon (Tn) null mutant and its parental wild type strain (Erdman) [36]. While the backgrounds of the *fadD26*::Tn and *hip* mutant differ (H37Rv derivative versus Erdman), comparison is possible since a common role for PDIM has been reported in these two strains [36], [37]. In both cases, the PDIM mutant produced more persisters than its parental strain (Fig 3A and 3B). Interestingly, a PDIM-transport protein *drrA* (Rv2936) H37Rv mutant [38], which produces PDIM but does not correctly localize it to the cell wall, did not have a *hip* phenotype (S2 Fig). This suggests that it is the biosynthesis of PDIM, rather than the lipid itself, that is responsible for the difference in drug tolerance. We noticed that the *fadD26*::Tn mutant exhibited a faster growth rate than the wild type (Erdman) (Fig 3B). While there was no significant difference in growth rate of KL2849 compared to wild type (mc^2^6020), the *hip* mutant had a slightly shorter generation time during early exponential growth (S5 Table). Taken together, these results suggest that the absence of PDIM production gives PDIM-null mutants a growth advantage. Since persister formation is growth stage-dependent, the changes in PDIM production may indirectly affect antibiotic tolerance by changing the timing of transition from exponential to stationary phase.

Genes and pathways that have previously been associated with persister formation were also of interest when analyzing the *M*. *tuberculosis hip* mutants. In *E*. *coli*, glycerol metabolism has been linked to persister formation [18], [20]. In the *hip* mutants, two confirmed *E*. *coli* persister gene candidates, glycerol-3-phosphate dehydrogenase *glpD1* (Rv2249c) and glycerol-3-phosphate acyltransferase *plsB2* (Rv2484c), were found to contain non-synonymous mutations. We tested whether overexpression of either *glpD1* or *plsB2*, confirmed by immunoblot, had an effect on persister formation but the results were negative (S3 Fig). There are several possible explanations for the lack of an effect on persisters by overexpression of these two genes. First, there are two copies of each of these genes in *M*. *tuberculosis*, which may provide functional genetic redundancy. Second, downregulation rather than overexpression may be required as in the case of *plsB* in *E*. *coli*. Both genes were significantly downregulated in various *hip* mutants, supporting this possibility (S6 Table). In addition to *glpD1* and *plsB2*, we found several mutations in glycerol kinase *glpK* (Rv3696c). In *E*. *coli*, a *glpK*-null mutant had no persister phenotype [18] therefore we did not pursue it further as a candidate. Genes involved in the TCA cycle have also been implicated in drug tolerance in *M*. *tuberculosis* [28], [39]. In the three representative *hip* mutants, isocitrate lyase *icl* (Rv0467) was significantly upregulated in stationary phase (S7 Table) while citrate synthase *gltA1* (Rv1131) contained a non-synonymous SNP and was upregulated in KL1117 (S6 Table).

We noted global changes in metabolism of the *hip* mutants by transcriptome analysis showing significant upregulation of genes associated with energy production, such as a possible oxidoreductase (Rv3742c), inorganic ion metabolism, such as rubredoxin *rubA* and *rubB* (Rv3250c-Rv3251c), and lactate dehydrogenase (Rv1872c) (S8 Table). Downregulation of two transcriptional regulators (Rv0273 and Rv3066) is of interest as they may control of the expression of multiple genes (S8 Table). Another candidate is probable asparagine synthase C *asnC* family transcriptional regulator (Rv2324). As a feast/famine regulatory protein, the *asnC* transcriptional regulator controls the expression of a large number of genes in response to stress or changes in the environment [40]. While there was no clear trend in differential expression of Rv2324 (S6 Table), multiple independent mutations occurred in the *hip* mutants in this gene as well as in the hypothetical protein (Rv2323c) directly upstream of it, which suggests an association with the *hip* phenotype.

### Characterization of clinical isolates with a *hip* phenotype

#### Longitudinal clinical isolate analysis

**Fig 4:**
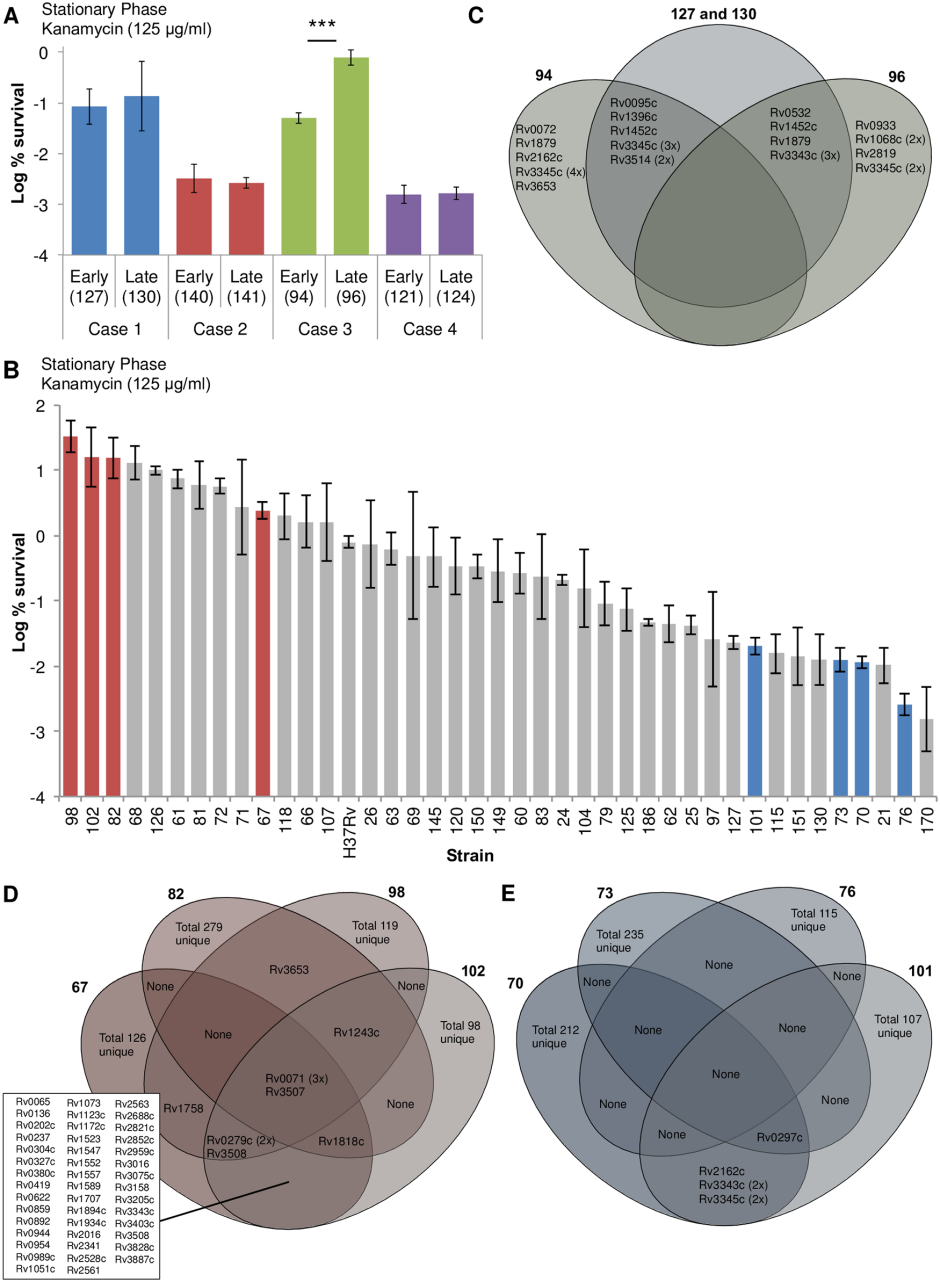
Characterization of clinical isolates. Longitudinal pairs of isolates from four cases of recalcitrant tuberculosis infection (A) or individual drug sensitive clinical isolates (B) were grown to stationary phase and treated with kanamycin (125 μg/ml) for 14 days and survival monitored by CFU counts. Genes containing non-synonymous SNPs differentiating the early and late isolate from Case 3 (C), unique to the four *hip* isolates (D), or unique the four low persister isolates (E) are presented. The values are an average of three biological replicates, error bars represent standard deviation, and stars represent significant difference (*p*-value < 0.001).

Repeated exposure of a mutagenized population to high doses of antibiotics enabled us to select for *hip* mutants of *M*. *tuberculosis in vitro*. We reasoned that in a clinical setting repeated treatment of patients with high doses of antibiotics might also select for *hip* mutants. To examine this possibility, we assessed persister levels of matched pairs of isolates from four longitudinal clinical tuberculosis cases. In each case, due to relapse or treatment failure, the patient had been treated for an extended period (S9 Table). The early and late isolates were collected at least 24 months apart and the pairs were matched based on identical high-resolution (24-loci) mycobacterial interspersed repetitive unit—variable number tandem repeat (MIRU-VNTR) genotyping patterns. Importantly, while some of the isolates were drug resistant (S9 Table), there was no change in MIC of the antibiotic used in the persister assay (S10 Table). To control for variation in growth rates among isolates, we performed the assay in stationary phase. When treated with kanamycin (125 μg/ml), one longitudinal pair (Case 3) exhibited a 10-fold increase in the level of persister formation from early to late (Fig 4A). This suggested that *hip* mutant selection could occur in clinical isolates of *M*. *tuberculosis*.

#### Screen of independent, drug-sensitive clinical isolates

In the longitudinal study, we observed large variation in persister levels among independent isolates (Fig 4A), which further supported the possibility that *hip* strains exist among clinical isolates. To examine this possibility, we screened a large panel of 39 independent, drug-sensitive clinical isolates (S9 Table). The isolates were all equally susceptible to the three antibiotics used in the persister assay (S11 Table). When treated in stationary phase with kanamycin (125 μg/ml), the level of persisters varied up to 10,000-fold among individual isolates (Fig 4B). Large variations in persister levels were also observed when the isolates were treated with moxifloxacin (20 μg/ml) or rifampicin (10 μg/ml) (S4 Fig). There was high correlation in isolate-specific persister level between kanamycin and moxifloxacin (r = 0.75, *p*-value < 0.01) but less correlation between kanamycin and rifampicin (r = 0.32, *p*-value < 0.05). To elucidate underlying genetic differences of strains that produce more persisters, we selected four high and low persister clinical isolates for further characterization.

#### Whole genome sequencing analysis of clinical isolates

Whole genome sequencing showed that the total number of non-synonymous SNPs between the clinical isolates and the reference strain (H37Rv) ranged from 156 to 854 with a mean of 709 per isolate (S12 Table). For the longitudinal pair of Case 3, we filtered the number of SNPs analyzed by eliminating any that occurred in Case 1, where there was no change in persister level (Fig 4C and S13 Table). Of the 28 non-synonymous SNPs identified, 23 occurred in proline-glutamate (PE) or proline-proline-glutamate (PPE) genes. Since PE/PPE genes are known to be highly variable in *M*. *tuberculosis*, we excluded them from further consideration as candidate genes involved in the *hip* phenotype. The remaining five included a glutamate transport transmembrane protein (Rv0072), a phosphate ABC transporter (Rv0933), and three hypothetical proteins (Rv0095c, Rv1879, and Rv2819c). For analysis of the panel of drug sensitive isolates, similar to our assessment of the *hip* mutants obtained *in vitro*, we looked for overlapping mutations. There were four identical non-synonymous SNPs that were unique to the *hip* isolates (Fig 4D and S14 Table). A possible maturase protein (Rv0071c) was the only non-PE/PPE gene that contained a common SNP in all four *hip* clinical isolates. There were no non-synonymous SNPs unique to all four low persister isolates, although one mutation in a PE/PPE gene (Rv0279c) occurred in three of them (Fig 4E and S14 Table). Next, we compared the high and low persister clinical isolates with the *hip* mutants obtained *in vitro*. Half of the 60 genes that possessed non-synonymous mutations in the *in vitro hip* mutants also contained mutations in the clinical isolates (S15 Table). Of those 30 genes, 12 contained non-synonymous mutations that were specific to at least one *hip* clinical isolate including a transcriptional regulator (Rv1395), *plsB2* (Rv2482c), phenolphthiocerol synthesis type-1 polyketide synthase *ppsD* (Rv2933), and mycoserosic acid synthase *mas* (Rv2940c). This comprehensive genomic comparison of *in vitro* mutants and clinical isolates points to several novel candidate genes that may play a role in persister formation.

#### Transcriptome analysis of persister cells from clinical *hip* isolates

**Fig 5:**
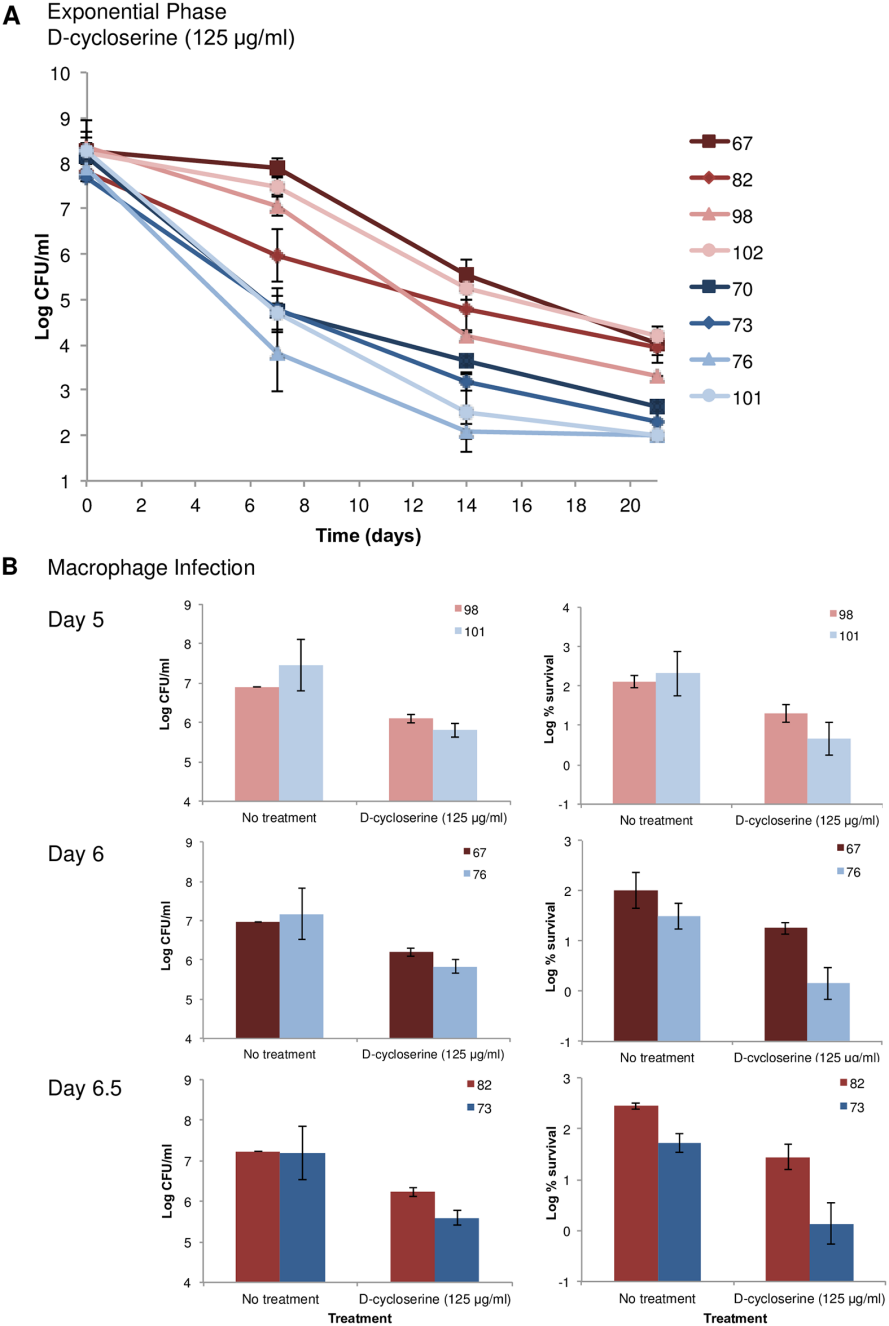
Antibiotic tolerance of *hip* and low persister clinical isolates. Eight individual clinical isolates were grown exponential phase and treated with D-cycloserine (125 μg/ml) for 21 days and survival monitored by CFU counts (A). Macrophages were infected with either a *hip* or a low persister clinical isolate for 12 hrs and then treated with D-cycloserine (125 μg/ml) for 5 to 6 days and bacterial survival was determined by lysing the macrophages and plating for CFU (B). The values are an average of three biological replicates for each sample and the error bars represent standard deviation.

To further elucidate the differences between the clinical *hip* and low persister isolates, we compared their transcriptome profiles. We note that the *in vitro* transcriptome of clinical isolates could be different from their transcriptome during infection. At the same time, we reasoned that the genetic changes leading to a higher probability for producing persisters and that are observed *in vitro*, will similarly manifest themselves *in vivo*. The most straightforward way to isolate persisters *in vitro* is by lysing a growing culture with a cell wall synthesis inhibitor and collecting surviving persisters by centrifugation [7]. D-cycloserine is an effective cell-lysing antibiotic of *M*. *tuberculosis* [41] and has been successfully used to isolate persisters [21]. Importantly, there was no difference in the D-cycloserine MIC among the eight clinical isolates (S11 Table). The *hip* isolates, treated in mid-exponential phase (OD_600_ 0.5) with D-cycloserine, produced more survivors than the low persister isolates (Fig 5A). The greatest difference between the *hip* and low persister isolates occurred at day 7 of treatment. We therefore collected samples at days 0 and 7 for transcriptome analysis. Comparative analysis between sample pairs of each isolate on day 0 and day 7 revealed that 13 genes were upregulated >4-fold in all four *hip* clinical isolates (S16 Table). Three of these genes, including a transcriptional regulator (Rv2989), a hypothetical protein (Rv1291c), and an amino acid cysteine synthase (Rv0848), were uniquely upregulated in the *hip* isolates while the other 10 were upregulated >4-fold in all of the low persister isolates (S17 Table). Upregulated genes in both groups include a heat shock protein (Rv0251c), catalase-peroxidase-peroxynitritase T *katG* (Rv1908c), *gltA1* (Rv1131), *clpC2* (Rv2667), and *glpD1* (Rv2249c). There were 16 genes downregulated >4-fold in all four *hip* isolates (S18 Table). The majority of these showed significantly higher expression in the *hip* isolates compared to the low persister isolates at day 0 (representing the bulk population), prior to antibiotic treatment. In the low persister isolates, genes that were downregulated >4-fold included many of those identified in the *hip* isolates including two resuscitation-promoting factors, *rpfC* (Rv1884c) and *rpfD* (Rv2389c) (S19 Table). Comparative analysis of *hip* versus low persister isolates at day 0 and day 7 revealed genes that were upregulated >4-fold including triacylglycerol synthase *tsg1* (Rv3130c), the devRS two-component system (Rv3132c-Rv3133c) and cation-transporting ATPase (Rv3743c) (S20 Table). Interestingly, Rv3743c is involved in energy metabolism and is directly adjacent to the most upregulated gene identified in the *in vitro hip* mutants, Rv3742c (S8 Table).

TA modules have previously been associated with antibiotic tolerance. We therefore assessed the clinical isolate persister transcriptomes for each of the 79 TA modules identified to date in *M*. *tuberculosis* [23]. There were over 34 TA modules upregulated in both the *hip* and low persister transcriptomes and 15 that were uniquely upregulated in *hip* isolates (S21 Table). Among the upregulated TA modules were the 10 identified in the previous *M*. *tuberculosis* H37Rv persister transcriptome [21], including *E*. *coli relE* homologue (Rv2866) that is known for its involvement in *M*. *tuberculosis* drug tolerance [24].

Of the genes identified from the longitudinal study, phosphate-transport ATP-binding protein ABC transporter *pstB* (Rv0933) was downregulated in all strains and glutamine-transport transmembrane protein ABC transporter (Rv0072) was dramatically downregulated (>400-fold) in the *hip* isolates compared to the low persister isolates (S22 Table). Of the clinical *hip* isolate mutations, the possible maturase gene (Rv0071) was also significantly downregulated in the *hip* compared to the low persister isolates (S22 Table). Of the *in vitro hip* mutant candidate genes identified by whole genome sequencing analysis, genes highly upregulated in both the *hip* and low persister isolates included *gltA1* (Rv1131), *glpD1* (Rv2249c), hypothetical protein (Rv2323c), and transcriptional regulator AsnC family (Rv2324) (S22 Table). Of the PDIM biosynthesis genes, *fadD26*, *ppsA* (Rv2931), and *mas* were significantly downregulated in both *hip* and low persister isolates while other PDIM genes showed variable expression (S23 Table). Of the 15 genes significantly differentially expressed in the *hip* mutants obtained *in vitro*, eight showed similar differential expression in the clinical *hip* compared to low persister isolates at day 0, prior to antibiotic treatment (S24 Table). Eight out of 15 genes highly expressed in H37Rv persister transcriptome [21] were upregulated in both *hip* and low persister isolate transcriptomes (S25 Table).

### Clinical *hip* isolates in an *ex vivo* model of *M*. *tuberculosis* infection

With reduced killing of the *hip* clinical isolates by bactericidal antibiotics in the *in vitro* persister assays, we next sought to determine whether these isolates would exhibit a survival advantage in an *ex vivo* model of tuberculosis infection. Murine macrophages were infected with either a *hip* or a low persister clinical isolate. Once the infection was established, cells were treated with two separate antibiotics. To reduce variability between the *in vitro* and *ex vivo* assays, kanamycin and D-cycloserine were used. Even with moderate intracellular killing activity [42], these two antibiotics killed the low persister isolates to a greater extent than the *hip* isolates. Survival of the *hip* isolates was significantly higher (*p*-value < 0.05) than the low persister isolates at the later time points with D-cycloserine treatment (Fig 5B). Similar data were obtained using a two-fold higher concentration of antibiotic (S5 Fig). These results point to a link between the *hip* phenotype and increased *ex vivo* drug tolerance.

## Discussion

**Fig 6:**
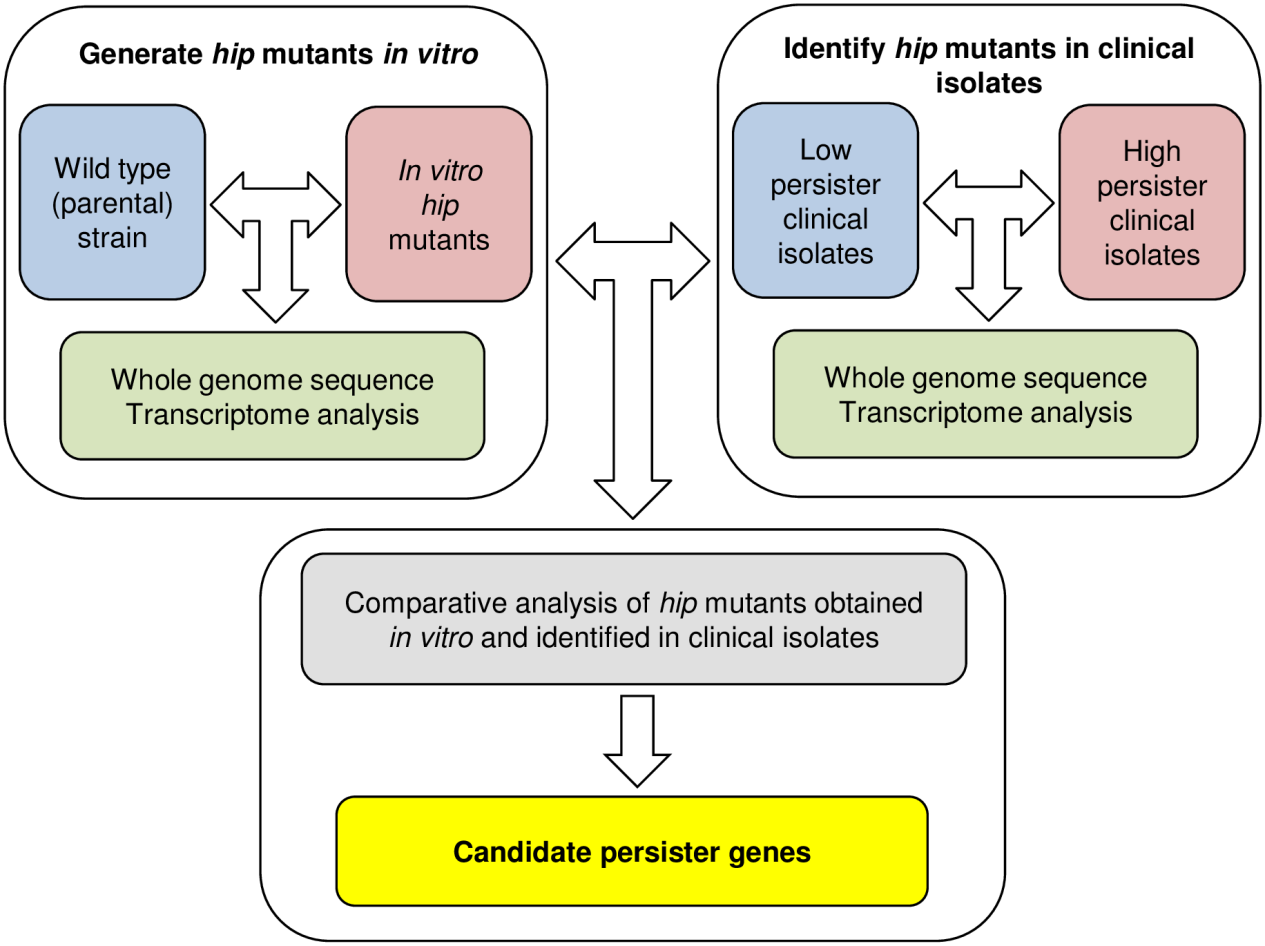
Summary of the study of *hip* mutants in *M*. *tuberculosis*. Schematic depicting the comparative analysis of *hip* mutants, generated *in vitro* and identified in clinical isolates, by whole genome sequencing and transcriptome analysis to identify candidate persister genes.

Persisters are thought to be responsible for the lengthy therapy of acute tuberculosis, and for the existence of the latent disease. In this study, we aimed to identify potential persister genes by studying high persister (*hip*) mutants (Fig 6). Repeated selection for cells surviving antibiotic treatment *in vitro* resulted in the formation of up to 1,000 times more persister cells by the *hip* mutants than by the parental wild type strain. Antibiotic therapy similarly exposes patients to periodic treatment with high levels of bactericidal drugs. Importantly, we also found *hip* mutants among human isolates, which links persisters to the clinical manifestation of disease.

Whole genome sequencing of *hip* mutants obtained *in vitro* identified several candidate persister genes. Multiple independent mutations were found within the PDIM biosynthetic operon. PDIM is an important cell wall lipid and virulence factor whose synthesis involves a large locus consisting of 13 genes. Repeated culture was reported to lead to a loss of PDIM; it is expensive to produce, and not required in vitro [37], [43], [44]. Indeed, PDIM-null mutants have a growth advantage [37]. We also find that PDIM mutants grow to a higher density. Since the frequency of persister formation is density dependent, the higher cell concentration of PDIM mutants could be responsible for their *hip* phenotype. Mutations in PDIM biosynthetic genes were also identified by whole genome sequencing of clinical isolates. While the *ppsD* (Rv2934) mutation in the *hip* clinical isolate 82 (S1132R) and *mas* (Rv2940c) mutation in *hip* isolate 102 (A195V) did not seem to be directly associated with any change in expression, transcriptome analysis showed significant downregulation of *fadD26*, *ppsA*, *mas*, *fadD28* (Rv2941), and transmembrane transport protein *mmpL7* (Rv2942) in various clinical isolates. The decreased expression of PDIM may allow *M*. *tuberculosis* to retain its virulence and at the same time increase the production of drug-tolerant persisters. The mechanism underlying the decreased expression of PDIM genes in *hip* mutants remains to be established.

Phospholipid biosynthesis has been associated with persister formation in *E*. *coli*. Mutations in *plsB2* (Rv2482c) and cdp-diacylglycerol pyrophospatase *cdh* (Rv2289), both involved in phospholipid biosynthesis were identified in *hip* mutants obtained *in vitro* and in clinical isolates. Mutations in *plsB2* were identified in *hip* mutant KL2826 (L221F), obtained *in vitro*, as well as in a *hip* clinical isolate 82 (R159H and D617H) and in a low persister clinical isolate 70 (R179Q and D328G). There was no obvious trend in differential expression of *plsB2* in the persister fraction from the clinical isolates, however, this gene was significantly downregulated in stationary phase compared to wild type (mc^2^6020) in several of the *hip* mutants obtained *in vitro* (KL2801, KL2849, KL1116, KL1130). Mutations in *cdh* were identified in *hip* mutant KL1120 (A122V) obtained *in vitro* as well as a *hip* clinical isolate 82 (K215I) and a low persister clinical isolate 76 (L233R). This gene was significantly downregulated in the persister fraction of the clinical isolates. Together this data further suggests that a decrease in phospholipid biosynthesis may be associated with persister formation in *M*. *tuberculosis*.

Besides phospholipid biosynthesis, alterations in other metabolic pathways are known to affect persister formation. For example, genes that inhibit lipid catabolism have been linked to drug tolerance [28]. Mutations in acyl-CoA dehydrogenase *fadE30* (Rv3560c), a gene involved in lipid degradation, were identified in a *hip* mutant KL2825 (R381W) obtained *in vitro* and in *hip* clinical isolate 82 (E34A) where it was found to be downregulated in the persister fraction. Changes in amino acid metabolism are also known to affect persistence [45]. Among the mutant genes differentiating the late isolate of our longitudinal study, a phosphate ABC transporter (Rv0933) and a glutamate ABC transporter (Rv0072) involved in amino acid metabolism, were identified. Importantly, the expression of Rv0072 was more than 400-fold lower in the *hip* isolates compared to the low persister isolates. Mutations in isoleucyl-tRNA synthetase *ileS* (Rv1536) were identified in *hip* mutant KL1134 (E728K) obtained *in vitro* and *hip* clinical isolate 82 (R39H). Also involved in amino acid biosynthesis and reported to be upregulated during Mg^2+^ starvation [46], this gene was upregulated in the persister fraction of clinical isolate 82. We also observed a mutation in *gltA1* (Rv1131) and its upregulation in a *hip* mutant obtained *in vitro* (KL1117). While there were no mutations in *gltA1* in the clinical isolates, this gene was significantly upregulated in the persister fraction of all isolates. Upregulation of *gltA1* has been linked to a metabolic shift in carbon away from the TCA cycle, directly affecting antibiotic tolerance [28].

Pathways not previously associated with persister formation were also identified in our study. A single non-synonymous mutation in maturase-like protein (Rv0071) was unique to all four *hip* clinical isolates (A118V). This gene was significantly downregulated in the *hip* clinical isolates. Little is known about RNA maturation enzymes in bacteria. They function like group II introns and may play a role in RNA editing [47]. In addition, mutations in the probable transcriptional regulator of the AsnC family (Rv2324) were identified in several *hip* mutants obtained *in vitro* (KL1090, KL1105, KL1116, KL1117). AsnC regulatory proteins are known as the ‘feast or famine’ proteins in bacteria and are involved in direct regulation of multiple genes in response to environmental signals [40]. While no mutations were found in the clinical isolates, this transcriptional regulator was significantly upregulated over 16-fold in the persister fraction of three *hip* clinical isolates (82, 98, 102). Another candidate identified was transcriptional regulator Rv1395, known to induce the expression of cytochrome P450 gene Rv1394 [48]. Mutations in Rv1395 were identified in *hip* mutant KL2801 (P208L) obtained *in vitro* and in *hip* clinical isolates 65 (I105M) and 82 (R220C). This gene was significantly upregulated in the persister fraction of all eight clinical isolates and as such it is an interesting candidate for further analysis.

Transcriptome analysis revealed an upregulation in TA module expression in the persister fraction of *M*. *tuberculosis* clinical isolates. Various TA modules induce drug tolerance in *M*. *smegmatis*, *M*. *tuberculosis*, and *E*. *coli* [7], [21], [23]. One of the most upregulated TA modules identified in our study was *HigBA1* (Rv1955-Rv1957). This is a tripartite module comprised of toxin-antitoxin-chaperone (TAC) where activation of the toxin likely requires unavailability of the chaperone and/or degradation of the antitoxin [23]. Interestingly, the antitoxin HigA1 (Rv1956) of this TAC is a putative regulator of the fatty acid metabolism gene cluster Rv2930-Rv2939, comprising PDIM biosynthetic genes including *fadD26* [49]. It is also capable or interacting with the stress response gene cluster Rv3249-Rv3252, including *rubA* and *rubB*, which were upregulated in the *hip* mutants obtained *in vitro*. While only one mutation was identified in this TAC module in our study (clinical isolate 73; Rv1957, D83N), the notable transcriptional profile and association with other persister gene candidates provides some evidence for a putative role in the *hip* phenotype. Moreover, we found that, just as with H37Rv [21], *clpC2* (Rv2667) was upregulated in the persister fraction of clinical isolates. Since proteases typically degrade antitoxins leading to toxin-mediated drug tolerance, this further supports a role for TA modules in *M*. *tuberculosis* persister formation.

The identification of *hip* mutants in longitudinal isolates of *C*. *albicans* [32] and *P*. *aeruginosa* [33] suggests that *hip* mutants may be a general feature of recalcitrant infectious diseases. Our identification of *hip* clinical isolates of *M*. *tuberculosis* supports this possibility. Interestingly, infection with the *hip* clinical isolate 67 resulted in relapse whereas infection with none of the low persister isolates did (S9 Table). Note that *hip* isolate 102 was isolated from a patient with a history of four repeated episodes of tuberculosis. The *hip* clinical isolates showed increased drug tolerance both *in vitro* and in an *ex vivo* model of tuberculosis. These findings are in agreement with a previous report of delayed killing *in vitro* of *M*. *tuberculosis* isolates from patients with relapse [50].

The results of this study reveal an overlap in genes and pathways we identified by transcriptome analysis of persisters and genomic analysis of *hip* mutants with those reported to be associated with persister formation, such as TA modules and carbon and amino acid metabolism. Our study also points to novel genes that may be unique to *M*. *tuberculosis* persisters, such as lipid biosynthesis and feast or famine regulation. Knowledge of *M*. *tuberculosis* persister cells will facilitate development of effective therapies for their eradication.

## Materials and Methods

### Strains, media and culture conditions

Bacterial strains used in this study are shown in Table 2. *M*. *tuberculosis* H37Rv double auxotroph strain mc^2^6020 [35] was used as the parental strain of the *in vitro hip* mutants. Clinical isolates included in this study were obtained from new tuberculosis cases and retreatment cases of subjects enrolled in a prospective longitudinal cohort study (ClinicalTrials.gov identifier, NCT00341601) at the National Masan Hospital (NMH) in the Republic of Korea. The institutional review boards of National Institute of Allergy and Infectious Diseases (NIAID) and NMH approved the study and all subjects gave written informed consent. H37Rv (American Type Culture Collection [ATCC] No. 27294) was used as the wild type strain in the clinical isolate experiments. PDIM mutant strains mc^2^3105 [36] and *drrA*::Tn [38] were kindly provided. Strains were grown in Middlebrook 7H9 Broth or on 7H10 Agar (Difco) medium supplemented with 10% oleic acid-albumin-dextrose-catalase (OADC) (Difco), 0.2–0.5% glycerol, either 0.05% Tween-80 (clinical isolate broth experiments) or 0.05% tyloxapol (*in vitro hip* mutant broth experiments), and required supplements for the double auxotroph mc^2^6020 [35] including 0.2% casamino acids (Amresco), pantothenic acid (24 μg/ml), and lysine (80 μg/ml). Freezer stocks were diluted 1:100 into 7H9 broth medium and grown in a shaking incubator at 37°C at 100 rpm. Minimal media was prepared as previously described [51] with 0.5% glycerol (vol/vol), 0.1% butyrate (wt/vol), or 0.1% propionate (wt/vol), 0.05% tyloxapol (vol/vol), pantothenic acid (24 μg/ml), and lysine (80 μg/ml). The clinical isolates were grown first on Lowenstein-Jensen slants and then transferred to 7H9 media prior to freezing stocks. To limit the introduction of genetic mutations due to *in vitro* growth conditions, the number of passages of strains from the original stock was limited to a maximum of five in accordance with ATCC recommendations. All chemicals were obtained from Sigma-Aldrich unless otherwise indicated. The antibiotics and their concentrations used in this study were streptomycin (10 μg/ml), rifampicin (1 μg/ml), kanamycin (50 μg/ml), ofloxacin (10 μg/ml), hygromycin (50 μg/ml), D-cycloserine (125 μg/ml), and moxifloxacin (20 μg/ml) (Waterstone Technology). Antibiotic stocks were prepared as recommended [52] and subsequent dilutions were made in 7H9 broth medium.

**Table 2:** Bacterial strains used in this study.

Strain Name	SRA Accession Number	Description	Parent Strain Name	Reference
mc^2^6020		H37Rv Δ*lysA* Δ*panCD*	H37Rv	[35]
KL2801	SRX014112	Hip mutant KL2801	mc^2^6020	This study
KL2802	SRX016225	Hip mutant KL2802	mc^2^6020	This study
KL2803	SRX014110	Hip mutant KL2803	mc^2^6020	This study
KL2825	SRX016226	Hip mutant KL2825	mc^2^6020	This study
KL2826	SRX016227	Hip mutant KL2826	mc^2^6020	This study
KL2827	SRX016228	Hip mutant KL2827	mc^2^6020	This study
KL2849	SRX014107	Hip mutant KL2849	mc^2^6020	This study
KL2850	SRX014911	Hip mutant KL2850	mc^2^6020	This study
KL2851	SRX014909	Hip mutant KL2851	mc^2^6020	This study
KL1090	SRX005187	Hip mutant HTS1090	mc^2^6020	This study
KL1105	SRX000677	Hip mutant HTS1105	mc^2^6020	This study
KL1116	SRX081440	Hip mutant HT1116	mc^2^6020	This study
KL1117	SRX081849	Hip mutant HT1117	mc^2^6020	This study
KL1120	SRX081404	Hip mutant HT1120	mc^2^6020	This study
KL1130	SRX081415	Hip mutant HT1130	mc^2^6020	This study
KL1134	SRX081412	Hip mutant HT1134	mc^2^6020	This study
KL1137	SRX081421	Hip mutant HT1137	mc^2^6020	This study
KL1170	SRX081442	Hip mutant HT1170	mc^2^6020	This study
p-*glpD1*		mc^2^6020 carrying pTET*glpD1*, Kan^R^	mc^2^6020	This study
p-*glpD1*-flag		mc^2^6020 carrying pTET*glpD1*-flag, Kan^R^	mc^2^6020	This study
p-*plsB2*		mc^2^6020 carrying pTET*plsB2*, Kan^R^	mc^2^6020	This study
p-*plsB2*-flag		mc^2^6020 carrying pTET*plsB2*-flag, Kan^R^	mc^2^6020	This study
*fadD26*::Tn (Erdman)		mc^2^3105, Erdman *fadD26*::Tn5370, Hyg^R^	Erdman	[36]
Wild type (Erdman)		Wild type	Erdman	[36]
Wild type (H37Rv)		H37Rv carrying JEB403, Kan^R^	H37Rv	[38]
*drrA*::Tn (H37Rv)		H37Rv *drrA*::Tn.1 carrying JEB403, Hyg^R^, Kan^R^	H37Rv	[38]
*drrA*::Tn + complement (H37Rv)		H37Rv *drrA*::Tn.1 carrying JEB*drrA*, Hyg^R^, Kan^R^	H37Rv	[38]
H37Rv		Wild type (ATCC 27294)	H37Rv	ATCC
21		Clinical isolate		ClinicalTrials.gov identifier, NCT00341601
24		Clinical isolate		ClinicalTrials.gov identifier, NCT00341601
25		Clinical isolate		ClinicalTrials.gov identifier, NCT00341601
26		Clinical isolate		ClinicalTrials.gov identifier, NCT00341601
57		Clinical isolate		ClinicalTrials.gov identifier, NCT00341601
60		Clinical isolate		ClinicalTrials.gov identifier, NCT00341601
61		Clinical isolate		ClinicalTrials.gov identifier, NCT00341601
62		Clinical isolate		ClinicalTrials.gov identifier, NCT00341601
63		Clinical isolate		ClinicalTrials.gov identifier, NCT00341601
66		Clinical isolate		ClinicalTrials.gov identifier, NCT00341601
67		Clinical isolate		ClinicalTrials.gov identifier, NCT00341601
68		Clinical isolate		ClinicalTrials.gov identifier, NCT00341601
69		Clinical isolate		ClinicalTrials.gov identifier, NCT00341601
70		Clinical isolate		ClinicalTrials.gov identifier, NCT00341601
71		Clinical isolate		ClinicalTrials.gov identifier, NCT00341601
72		Clinical isolate		ClinicalTrials.gov identifier, NCT00341601
73		Clinical isolate		ClinicalTrials.gov identifier, NCT00341601
76		Clinical isolate		ClinicalTrials.gov identifier, NCT00341601
79		Clinical isolate		ClinicalTrials.gov identifier, NCT00341601
81		Clinical isolate		ClinicalTrials.gov identifier, NCT00341601
82		Clinical isolate		ClinicalTrials.gov identifier, NCT00341601
83		Clinical isolate		ClinicalTrials.gov identifier, NCT00341601
94		Clinical isolate		ClinicalTrials.gov identifier, NCT00341601
96		Clinical isolate		ClinicalTrials.gov identifier, NCT00341601
97		Clinical isolate		ClinicalTrials.gov identifier, NCT00341601
98		Clinical isolate		ClinicalTrials.gov identifier, NCT00341601
101		Clinical isolate		ClinicalTrials.gov identifier, NCT00341601
102		Clinical isolate		ClinicalTrials.gov identifier, NCT00341601
104		Clinical isolate		ClinicalTrials.gov identifier, NCT00341601
107		Clinical isolate		ClinicalTrials.gov identifier, NCT00341601
115		Clinical isolate		ClinicalTrials.gov identifier, NCT00341601
118		Clinical isolate		ClinicalTrials.gov identifier, NCT00341601
120		Clinical isolate		ClinicalTrials.gov identifier, NCT00341601
121		Clinical isolate		ClinicalTrials.gov identifier, NCT00341601
124		Clinical isolate		ClinicalTrials.gov identifier, NCT00341601
125		Clinical isolate		ClinicalTrials.gov identifier, NCT00341601
126		Clinical isolate		ClinicalTrials.gov identifier, NCT00341601
127		Clinical isolate		ClinicalTrials.gov identifier, NCT00341601
130		Clinical isolate		ClinicalTrials.gov identifier, NCT00341601
140		Clinical isolate		ClinicalTrials.gov identifier, NCT00341601
141		Clinical isolate		ClinicalTrials.gov identifier, NCT00341601
145		Clinical isolate		ClinicalTrials.gov identifier, NCT00341601
149		Clinical isolate		ClinicalTrials.gov identifier, NCT00341601
150		Clinical isolate		ClinicalTrials.gov identifier, NCT00341601
151		Clinical isolate		ClinicalTrials.gov identifier, NCT00341601
170		Clinical isolate		ClinicalTrials.gov identifier, NCT00341601
186		Clinical isolate		ClinicalTrials.gov identifier, NCT00341601

### Mutant strains and plasmid construction

Chemical mutagenesis was performed as previously described [53] with modifications. Strain mc^2^6020 was grown to exponential phase with an optical density at 600 nm (OD_600_) of 0.8 and washed two times with equal volumes of minimal A buffer (K_2_HPO_4_ 10.5 mg/ml, KH_2_PO_4_ 4.5 mg/ml, [NH_4_]_2_SO_4_ 1 mg/ml, [C_6_H_5_Na_3_O_7_]_2_H_2_O 0.5 mg/ml). Bacterial cells were resuspended in an equal volume of minimal A buffer. The cultures were split into 20 ml aliquots, 300 μl of ethyl methanesulfonate was added, and cultures were incubated on a shaker (100 rpm) at 37°C for 60 min. Mutagenized cells were washed two times with equal volumes of minimal A buffer and resuspended in 220 ml of 7H9 medium in 1,300 ml roller bottles (Corning). The roller bottles were incubated on a roller platform (6 rpm) at 37°C for one week until the culture reached stationary phase. Cultures were then diluted 1:100 into 40 ml of 7H9 medium, grown to exponential phase (OD_600_ 0.8) and then treated with a combination of streptomycin (10 μg/ml) and rifampicin (1 μg/ml) for one week. Cells were washed once with an equal volume of phosphate buffered saline (PBS) and resuspended in 40 ml of 7H9 medium. Before and after the antibiotic challenges, samples were taken for bacterial cell enumeration. The enrichment procedure was repeated. The washed cells were grown again to stationary phase, diluted 1:100, grown to exponential phase, and treated for one week using the same antibiotic combination. After the fourth round of enrichment individual strains were separated from the mutagenized populations by isolating individual colonies for further characterization.

Overexpression plasmids were constructed by cloning candidate genes using Phusion^®^ High-Fidelity DNA Polymerase (New England Biolabs) with the primers listed in S1 Table. Cloned products were ligated into the Gateway^®^ Cloning system (Invitrogen) and then transferred to an expression vector (pTET) containing a tetracycline inducible mycobacterial promoter [54]. Purified plasmids were transformed into *M*. *tuberculosis* mc^2^6020 and selected for using kanamycin (50 μg/ml). Overexpression strains were induced 72 hrs prior to antibiotic challenge by addition of anhydrous tetracycline (aTc) (100 ng/ml) to the media.

### Persister assay

To determine the level of persister formation in various strains and under various conditions, antibiotic treatments were performed in biological triplicate as previously described [21]. For stationary phase treatments, the initial two-week-old cultures were diluted 1:100 and grown for an additional two weeks prior to antibiotic treatment. For persister assays with the clinical isolates, freezer stocks were diluted 1:10 into broth media and grown for three weeks to stationary phase. Clinical isolate cultures were then diluted 1:100 into fresh broth media and grown for four to five days for exponential phase challenge or for three weeks for stationary phase challenge. Samples were plated at selected time points and bacterial survival was monitored by enumeration of colony forming units (CFU). The Student’s T-test was used to determine significant differences and Spearman’s Rank-order test was used to assess correlation.

### Growth rate determination

*Hip* mutant and wild type (mc^2^6020) strains were grown in triplicate in standard 7H9 liquid media and samples were taken at the indicated time points for cell enumeration by plating for CFU from which the generation time was calculated [55]. The data represent an average of three biological replicates plus or minus the standard deviation.

### Drug resistance assays

The drug sensitivity testing (DST) of clinical isolates was performed on solid media with absolute concentrations of first- and second-line anti-tuberculosis drugs. The anti-tuberculosis drugs that were tested included isoniazid (INH/H), rifampicin (RIF/R), streptomycin (SM/S), ethambutol (EMB/E), kanamycin (KM/K), capreomycin (CPM), prothionamide (PTH/T), cycloserine (CS/C), para-aminosalicylic acid (PAS/P), ofloxacin (OFX/O), moxifloxacin (MOX), amikacin (AMK), levofloxacin (LEV/Lf), rifabutin (RBU), and pyrazinamide (PZA/Z). The testing was carried out at the NMTH and the NIH according to standard guidelines for DST.

The MIC testing was liquid-based using the micro-plate based Alamar Blue assay as described [56] with minor modifications. Cultures was grown to early exponential phase (OD_600_ 0.3) and diluted to 1:100 for the assay. Antibiotics were serially diluted in 96-well plates and equal volumes of diluted bacterial cultures were added. The plates were sealed with Breathe-Easy^®^ (3M Company) and incubated at 37°C. After four days, 20 μl of Alamar Blue (Serotec) was added to each well and the plates were resealed and incubated for 24 to 48 hrs. The colors of all wells were recorded after visual inspection and absorbance measurement using the Synergy HT microplate reader (BioTek) with excitation and emission wavelengths of 530 nm and 590 nm, respectively.

### Total lipid analysis by thin layer chromatography

Total lipids were extracted by centrifugation of bacterial cells (4,000 rpm, 15 min) at room temperature. Pellets were resuspended in 5 ml of 2:1 CHCl_3_:MeOH and transferred to a clean glass centrifuge tube with a Teflon-lined cap. To the glass tube, 25 ml of CHCl_3_:MeOH (2:1) was added and lipids were extracted for at least 1 hr on an orbital rocker. Tubes were centrifuged (2,000 rpm, 15 min) at room temperature and supernatants were transferred to clean glass tubes. Pellets were resuspended in 30 mL of CHCl_3_:MeOH (1:1) and lipids extracted as above. Pellets were resuspended for a final time in 30 mL of CHCl_3_:MeOH (1:2). The organic phases were combined and evaporated using a rotovap. Extracted lipids were dissolved in CHCl_3_:MeOH (1:1) and 150 μg of each sample were spotted onto a 20 x 20 cm silica glass plate (84101, Scientific Adsorbents). Petroleum either:diethyl ether (90:10) was used as the solvent and the revelation solution consisted of 8% H_3_PO_4_ (vol/vol) and 3% Cu Acetate (wt/vol) in water. Plates were sprayed and heated in an oven to 160°C.

### Genomic DNA extraction and whole genome sequencing

Genomic DNA was isolated as previously described [57]. The concentration of genomic DNA was measured using a NanoDrop spectrophotometer (Thermo Scientific) and stored at -20°C. The Illumina paired-end reads design was used for whole genome sequencing of genomic DNA from the *hip* mutant and wild type (mc^2^6020) strains. All sequencing was performed at the Broad Institute (Cambridge, MA). Illumina fragment libraries were generated as previously described [58] with the following modifications. For each sample, 100 ng of genomic DNA was sheared to 200 bp in size using a Covaris LE220 instrument (Covaris, MA) with the following parameters: temperature: 7–9°C; duty cycle: 20%; intensity: 5; cycles per burst: 200; time: 90 sec; shearing tubes: Crimp Cap microTUBES with AFA fibers (Covaris, MA). DNA fragments were end repaired, 3′ adenylated, ligated with indexed Illumina sequencing adapter, and PCR enriched, as previously described [59]. The resulting Illumina fragment sequencing libraries were normalized and were size selected to contain inserts of 180 bp ± 3% in length using a Pippen Prep system (Sage Science, MA) following the manufacturer’s recommendations. Sequencing coverage is presented (S2 Table) and the data is available at the National Center for Biotechnology Information (NCBI) sequence read archive (SRA) under BioProject PRJNA38649. Sequenced strains were aligned to the fully assembled H37Rv library. The library name was Solexa-16183, source was genomic, selection was random, and layout was paired with a 5’3’-3’5’ orientation, a nominal length of 166, and a nominal standard deviation of 34.33. Coding single nucleotide polimorphisms (SNPs) as well as insertions and deletions that were identified between the *hip* mutants and the H37Rv reference assembly and that did not occur in the parental wild type strain (mc^2^6020) were classified as mutations. For analysis of the clinical isolates, we considered any coding SNP identified between each isolate and the H37Rv reference strain as a mutation.

### Transcriptome analysis

In collaboration with the Broad Institute (Cambridge, MA), Affymetrix microarray technology was used for conducting the transcriptome analysis of the *in vitro* generated *hip* mutant strains. Total RNA was extracted as previously described [21] from stationary phase cultures that were grown for 14 days from a 1:100 inoculum. Quality control of microarray data in our study was measured with RNA degradation and box plots. RNA degradation starts from the 5’ end to 3’ end so 5’ end probes show lower intensities than the 3’ end probes. RNA degradation plots measure this trend and a high slope indicates degradation. In this study RNA degradation was minimal. A boxplot is a tool to summarize intensity distributions and indicate if there are any sample outliers. Differences in amplification or labeling tend to cause these outliers. Our study did not show any outliers. Statistical analysis of the data was performed using the Bioconductor program and oneChannelGUI package [60] available through the R Project for Statistical Computing (www.r-project.org). Data is presented as log2 fold change. The microarray data have been deposited in NCBI’s Gene Expression Omnibus (GEO) [61] and are accessible through GEO Series accession number GSE55647.

For transcriptome analysis of the clinical isolates, cultures were grown from a 1:100 inoculum to mid-exponential phase (OD_600_ 0.5) and then treated with D-cycloserine (125 μg/ml). Culture samples were collected at Day 0 (2 ml) and Day 7 (40 ml) of treatment for RNA isolation as previously described [21]. Strand-specific RNAseq libraries were created by the Broad Technology Labs specialized service facility as described [62]. Briefly, 48 RNA samples were fragmented, and RNA 3’ ends were tagged with a DNA oligonucleotide containing a sample barcode and a partial 5’ Illumina adapter. Resulting barcoded RNAs were then pooled and subjected to rRNA depletion with RiboZero (Epicentre), cDNA synthesis and ligation to a second oligonucleotide containing a partial Illumina 3’ adapter. A second barcode specific to this pool was then added by amplification with full-length barcoded Illumina adapter primers, yielding a single strand-specific sequence-ready pooled RNA-seq library. The pool of 48 Illumina RNAseq libraries was quantified using qPCR (KAPA Biosystems) and sequenced with 76 base paired-end reads across 4 lanes using an Illumina HiSeq 2000 sequencer (Illumina) running v3 SBS chemistry. Sequence data were processed and demultiplexed using the Picard analysis pipeline (http://picard.sourceforge.net). Reads were aligned to the genome of *M*. *tuberculosis* H37Rv (RefSeq NC_000962) using BWA [63] version 5.9. Gene annotations were obtained from RefSeq and Rfam [64]. The overall fragment coverage of genomic regions corresponding to features such as ORFs and rRNAs was conducted as described [65]. RNAseq coverage metrics are presented (S3 Table). Differential expression analysis was conducted using DESeq [66]. Data is presented as log2 fold change. The RNAseq data are accessible through GEO Series accession number GSE62025.

### Immunoblot analysis

Flag-tagged proteins were visualized by immunoblot analysis. Proteins were extracted from bacterial cells by centrifugation (11,000 rpm, 15 min), followed by resuspension of the pellet in 250 μl of Bacterial Protein Extraction Regent (Thermo Scientific Pierce). Samples were placed in a bead beater tube with Lysing matrix B (MP Biochemicals), and disrupted by bead beating using FastPrep-24 (MP Biochemicals) for 45 sec at the power setting of 6. Proteins were separated on NuPAGE Novex 4–12% Bis-Tris Gels (Invitrogen) and transferred to nitrocellulose membranes. Monoclonal ANTI-FLAG^®^ M1 antibody produced in mouse (F30401; Sigma) was used at a dilution of 1:1,000. Membranes treated with the WesternBreeze^®^ Chemiluminescent Kit, anti-mouse (Invitrogen) according to the manufacturer’s instructions. Blots were developed with an X-ray developing system.

### Macrophage infections

The murine leukemic monocyte macrophage cell line RAW264.7 (ATCC) was used for all macrophage infections. Macrophages were propagated in Dulbecco’s Modification of Eagle’s Media with high glucose (Invitrogen) with 10% Fetal Bovine Serum, 2 mM L-glutamine, 1 mM sodium pyruvate, 10 mM HEPES, and 1% penicillin/streptomycin (optional) at 37°C with 5% CO_2_. Cells were grown to confluence and then seeded in 6-well plates at a concentration of 1 x 10^6^ cells per well and allowed to adhere to the surface of the plate overnight at 37°C with 5% CO_2_. Bacteria were grown to early exponential phase (OD_600_ 0.3) in 40 ml of 7H9 broth and collected by centrifugation (4,000 rpm, 10 min). Cells were resuspended in 5 ml of macrophage media (without penicillin/streptomycin), sonicated three times (5 sec) at amplitude 4 on the Misonix Ultrasonic Liquid Processor S-4000 (Qsonica), and vortexed three times (5 sec). The volume was brought to 40 ml with macrophage media (without penicillin/streptomycin) and the remaining clumps were allowed to settle for 15 min after which the top 35 ml was transferred to a new tube. The OD_600_ was measured and adjusted to 0.08 (~1 x 10^7^ CFU/ml). Bacteria were deposited in the 6-well plates containing macrophages at a multiplicity of infection of 10: 1 (bacteria: macrophages) for 12 hrs after which the extracellular bacteria were washed away five times and fresh media with or without antibiotics was added. At selected time points macrophages were lysed with 0.5% Triton-100 in PBS, serially diluted in PBS, and plated on 7H10 media for bacterial cell enumeration.

## Supporting Information

S1 Fig Thin layer chromatography of total lipid extracts. Total lipid extract (150 μg) from each strain was spotted on the plate along with a DIM-A standard and run in petroleum ether / diethyl ether (90/10) solvent. Spots were visualized by treating the plate with H_3_PO_4_ (8% v/v), Cu Acetate (3% v/v) and heat (140–160°C). DIM-A is present in the wild type strain (mc^2^6020) and *hip* mutant KL1116 but absent in all of the other *hip* mutant strains.
(TIF)

S2 Fig Persister assay of *drrA*::Tn PDIM mutant. The *drrA*::Tn mutant and complemented strain along with wild type (H37Rv) were grown to either exponential or stationary phase and treated with streptomycin (10 μg/ml) and rifampicin (1 μg/ml) for 14 days. Survival was monitored by CFU counts. The values are an average of three biological replicates and error bars represent standard deviation.
(TIF)

S3 Fig Overexpression of candidate persister genes *glpD1* and *plsB2*. Overexpression strains were induced with aTc (100 ng/μl) 72 hrs prior to antibiotic treatment. Induced overexpression of *glpD1* (A) and *plsB2* (B) resulted in no change in level of persister formation in either exponential or stationary phase. Immunoblot analysis confirms overexpression (right). The values are an average of three biological replicates and error bars represent standard deviation.
(TIF)

S4 Fig Characterization of persister level in clinical isolates. Individual drug sensitive clinical isolates were treated in stationary phase with moxifloxacin (20 μg/ml) (A) or rifampicin (10 μg/ml) (B) for 14 days and bacterial survival was determined plating for CFU. The values are an average of three biological replicates and error bars represent standard deviation.
(TIF)

S5 Fig Antibiotic tolerance of *hip* and low persister clinical isolates during macrophage infection. Murine macrophages were infected with either a *hip* or a low persister clinical isolate for 12 hrs and then treated with either kanamycin (250 μg/ml) or D-cycloserine (250 μg/ml) for up to 6 days. Bacterial survival was determined by plating for CFU after lysing the macrophages. The values are an average of three biological replicates for each sample and the error bars represent standard deviation.
(TIF)

S1 Table Oligonucleotides used in this study. (TIF)

S2 Table Whole genome sequencing coverage metrics. (TIF)

S3 Table RNAseq coverage metrics. (TIF)

S4 Table Minimum inhibitory concentration of antibiotics for wild type (mc^2^6020) and *in vitro hip* mutant strains. (TIF)

S5 Table Generation time (hrs) of wild type (mc^2^6020) and *in vitro hip* mutant strains under normal growth conditions. (TIF)

S6 Table Stationary phase gene expression of *hip* mutant versus wild type (mc^2^6020) of genes identified by whole genome sequencing of the 12 independent *in vitro hip* mutants. (TIF)

S7 Table Differential expression of top upregulated genes of three independent *in vitro* hip mutants (KL2801, KL2925, KL2849) versus wild type (mc^2^6020) in stationary phase. (TIF)

S8 Table Differential expression of 12 independent *in vitro hip* mutants versus wild type (mc^2^6020) in stationary phase. (TIF)

S9 Table Clinical isolate treatment history and drug resistance profile. (TIF)

S10 Table Minimum inhibitory concentration of kanamycin for longitudinal clinical isolates and H37Rv. (TIF)

S11 Table Minimum inhibitory concentration of antibiotics for clinical isolates and H37Rv. (TIF)

S12 Table Total number of non-synonymous SNPs in clinical isolates compared to reference strain H37Rv. (TIF)

S13 Table Non-synonymous SNPs differences between longitudinal isolates of Case 3 (94 and 96) compared to Case 1 (127 and 130). (TIF)

S14 Table Non-synonymous mutations unique to *hip* or low persister clinical isolates. (TIF)

S15 Table Non-synonymous SNPs in the clinical isolates that occur in the same genes as mutations in the *in vitro hip* mutants. (TIF)

S16 Table Genes upregulated (>4-fold) in all four *hip* clinical isolates. (TIF)

S17 Table Genes upregulated (>4-fold) in all four low persister clinical isolates. (TIF)

S18 Table Genes downregulated (>4-fold) in all four *hip* clinical isolates. (TIF)

S19 Table Genes downregulated (>4-fold) in all four low persister clinical isolates. (TIF)

S20 Table Genes upregulated (>4-fold) in *hip* versus low persister clinical isolates. (TIF)

S21 Table Clinical isolate transcriptome analysis of *M*. *tuberculosis* TA module genes. (TIF)

S22 Table Clinical isolate transcriptome analysis of candidate genes identified through whole genome sequencing. (TIF)

S23 Table Clinical isolate transcriptome analysis of PDIM biosynthetic operon genes. (TIF)

S24 Table Clinical isolate transcriptome analysis of genes differentially expressed in stationary phase *in vitro hip* mutants. (TIF)

S25 Table Clinical isolate transcriptome analysis of genes upregulated (>4-fold) in H37Rv persister cells. (TIF)
