# Supplementary figures and images for: High Persister Mutants in Mycobacterium tuberculosis
Source: PLoS One. 2016 May 13;11(5):e0155127. doi: 10.1371/journal.pone.0155127 (PMC4866775; doi:10.1371/journal.pone.0155127)

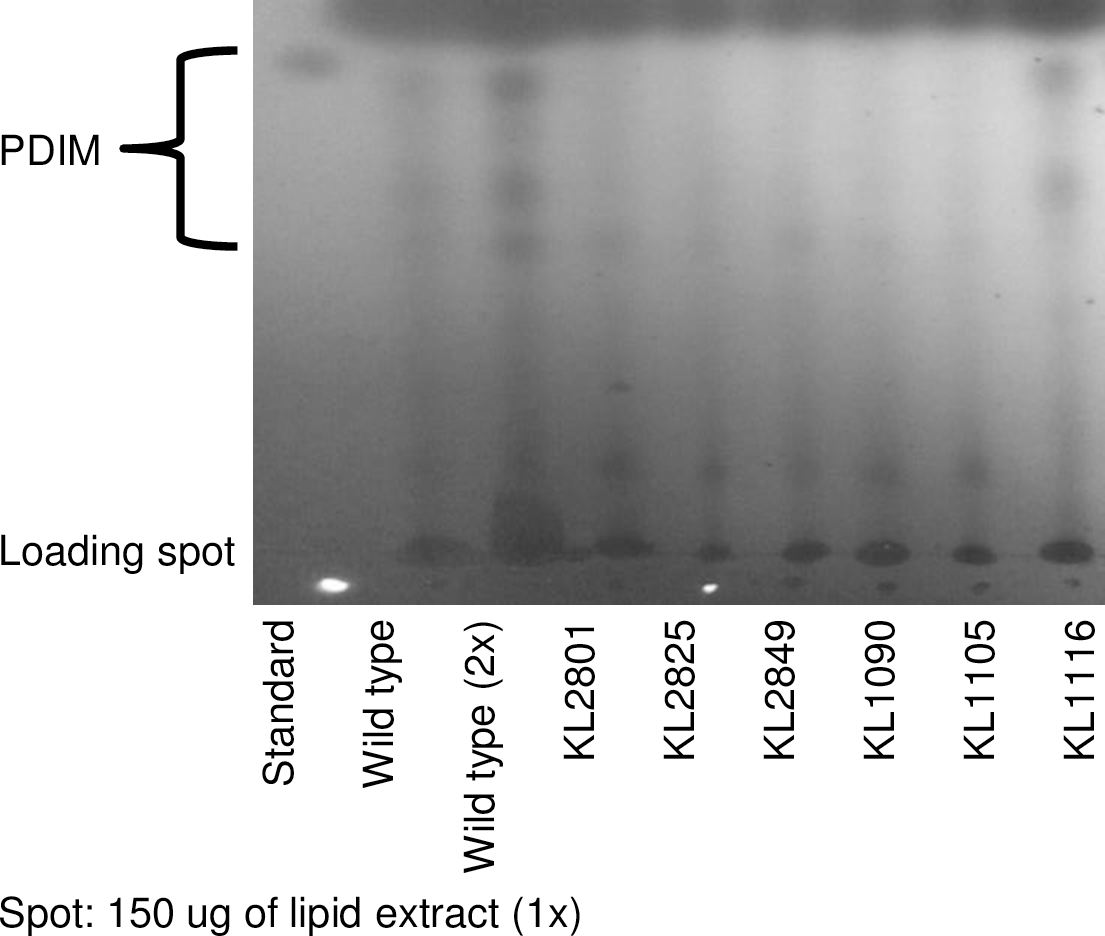

Supplement: S1 Fig — Total lipid extract (150 μg) from each strain was spotted on the plate along with a DIM-A standard and run in petroleum ether / diethyl ether (90/10) solvent. Spots were visualized by treating the plate with H3PO4 (8% v/v), Cu Acetate (3% v/v) and heat (140–160°C). DIM-A is present in the wild type strain (mc26020) and hip mutant KL1116 but absent in all of the other hip mutant strains. (TIF) [file pone.0155127.s001.tif]

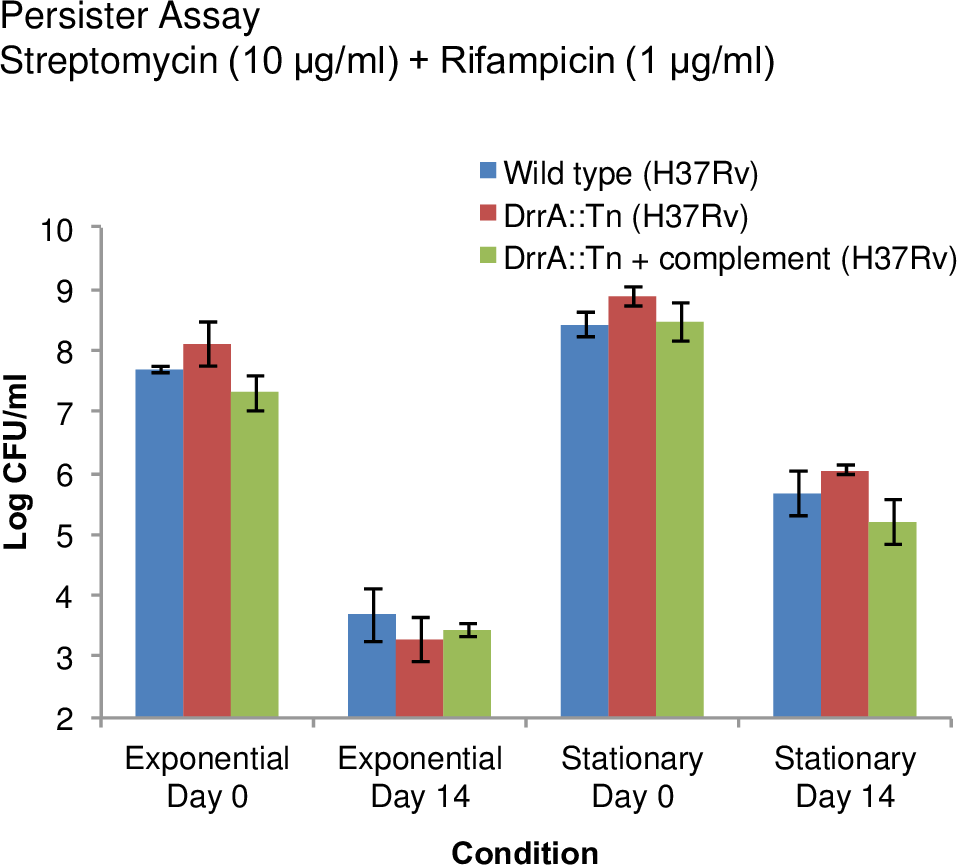

Supplement: S2 Fig — The drrA::Tn mutant and complemented strain along with wild type (H37Rv) were grown to either exponential or stationary phase and treated with streptomycin (10 μg/ml) and rifampicin (1 μg/ml) for 14 days. Survival was monitored by CFU counts. The values are an average of three biological replicates and error bars represent standard deviation. (TIF) [file pone.0155127.s002.tif]

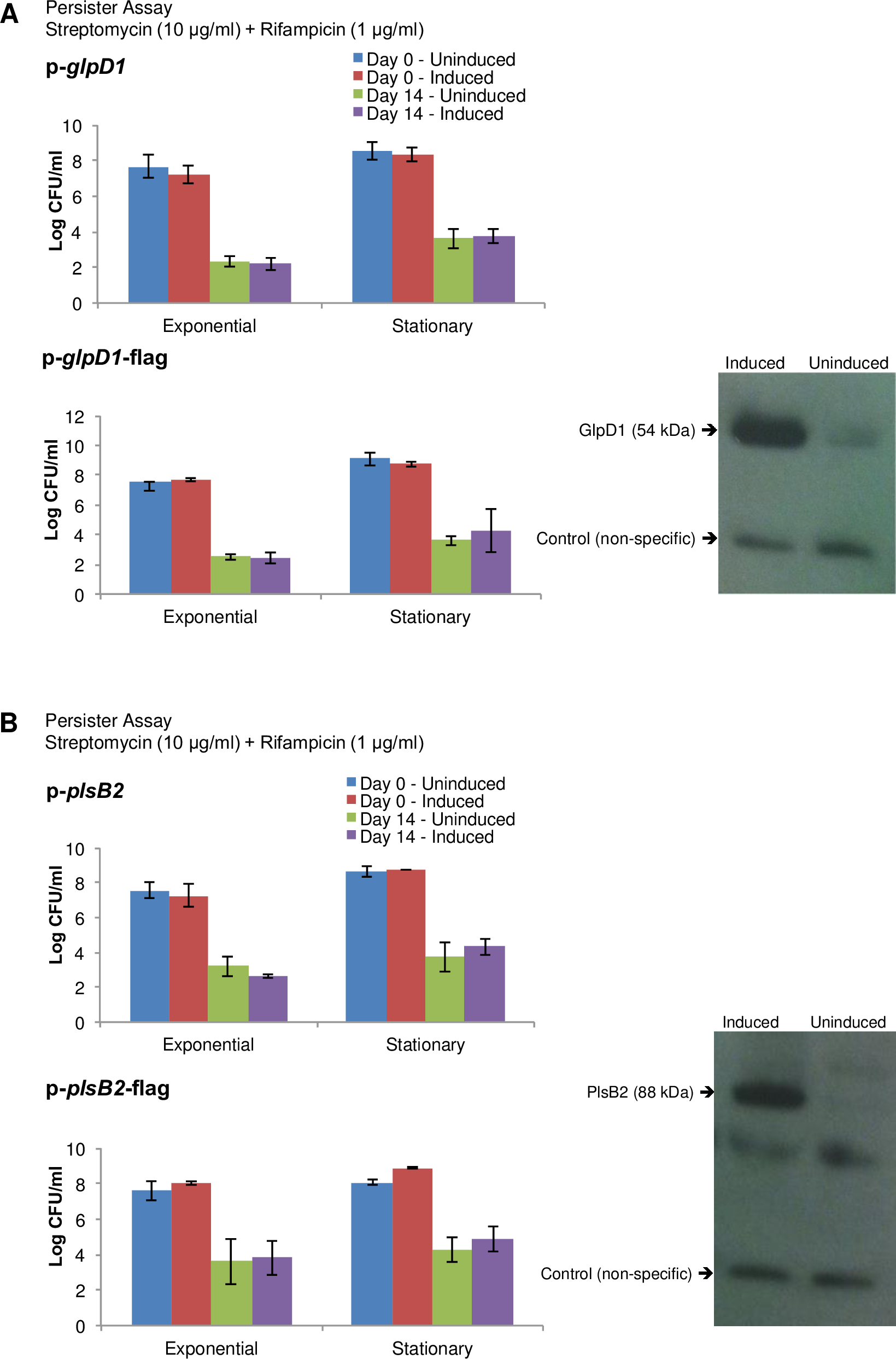

Supplement: S3 Fig — Overexpression strains were induced with aTc (100 ng/μl) 72 hrs prior to antibiotic treatment. Induced overexpression of glpD1 (A) and plsB2 (B) resulted in no change in level of persister formation in either exponential or stationary phase. Immunoblot analysis confirms overexpression (right). The values are an average of three biological replicates and error bars represent standard deviation. (TIF) [file pone.0155127.s003.tif]

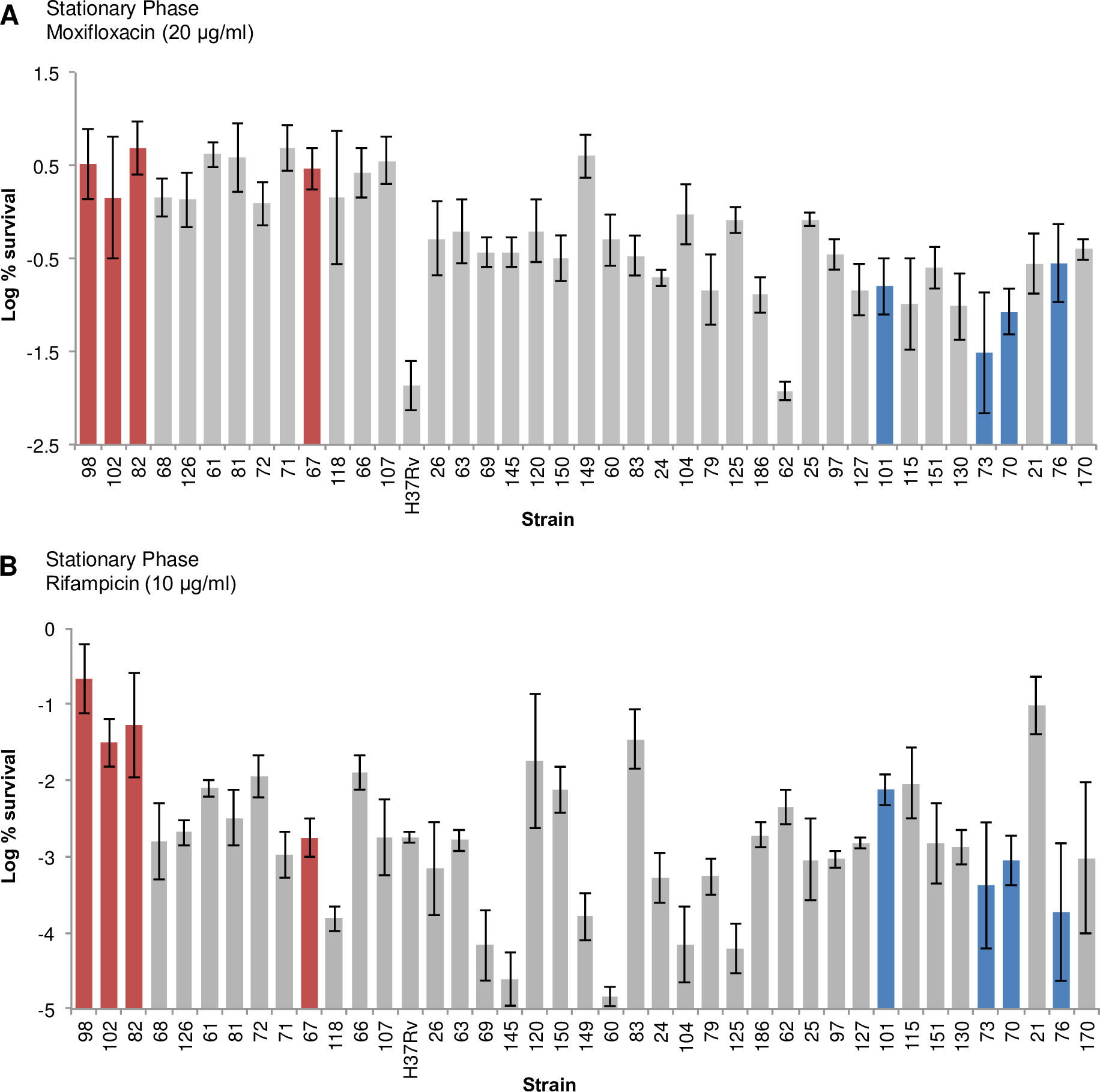

Supplement: S4 Fig — Individual drug sensitive clinical isolates were treated in stationary phase with moxifloxacin (20 μg/ml) (A) or rifampicin (10 μg/ml) (B) for 14 days and bacterial survival was determined plating for CFU. The values are an average of three biological replicates and error bars represent standard deviation. (TIF) [file pone.0155127.s004.tif]

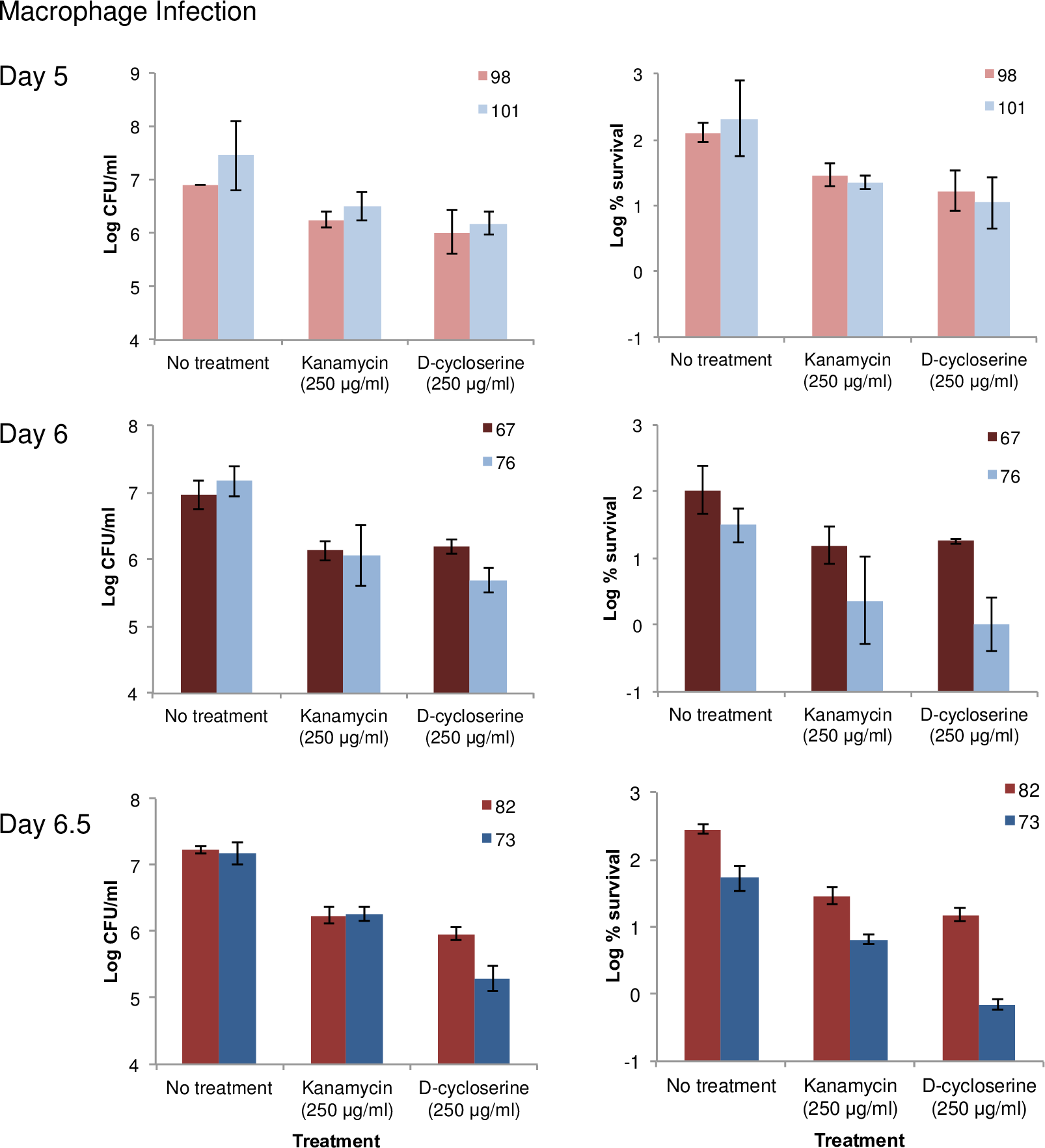

Supplement: S5 Fig — Murine macrophages were infected with either a hip or a low persister clinical isolate for 12 hrs and then treated with either kanamycin (250 μg/ml) or D-cycloserine (250 μg/ml) for up to 6 days. Bacterial survival was determined by plating for CFU after lysing the macrophages. The values are an average of three biological replicates for each sample and the error bars represent standard deviation. (TIF) [file pone.0155127.s005.tif]

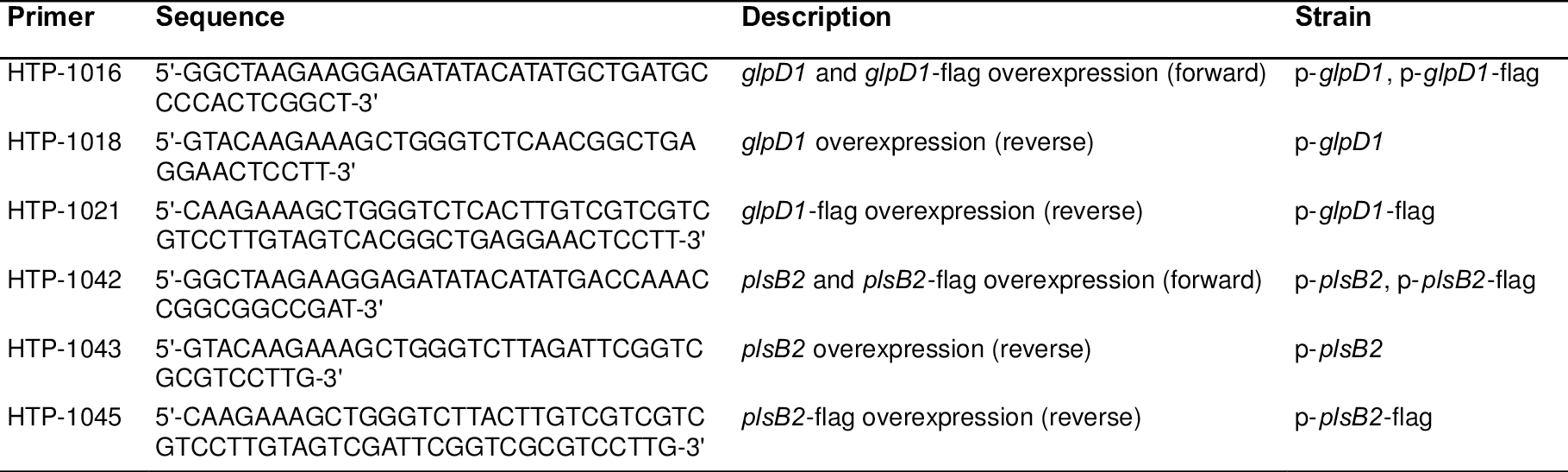

Supplement: S1 Table — (TIF) [file pone.0155127.s006.tif]

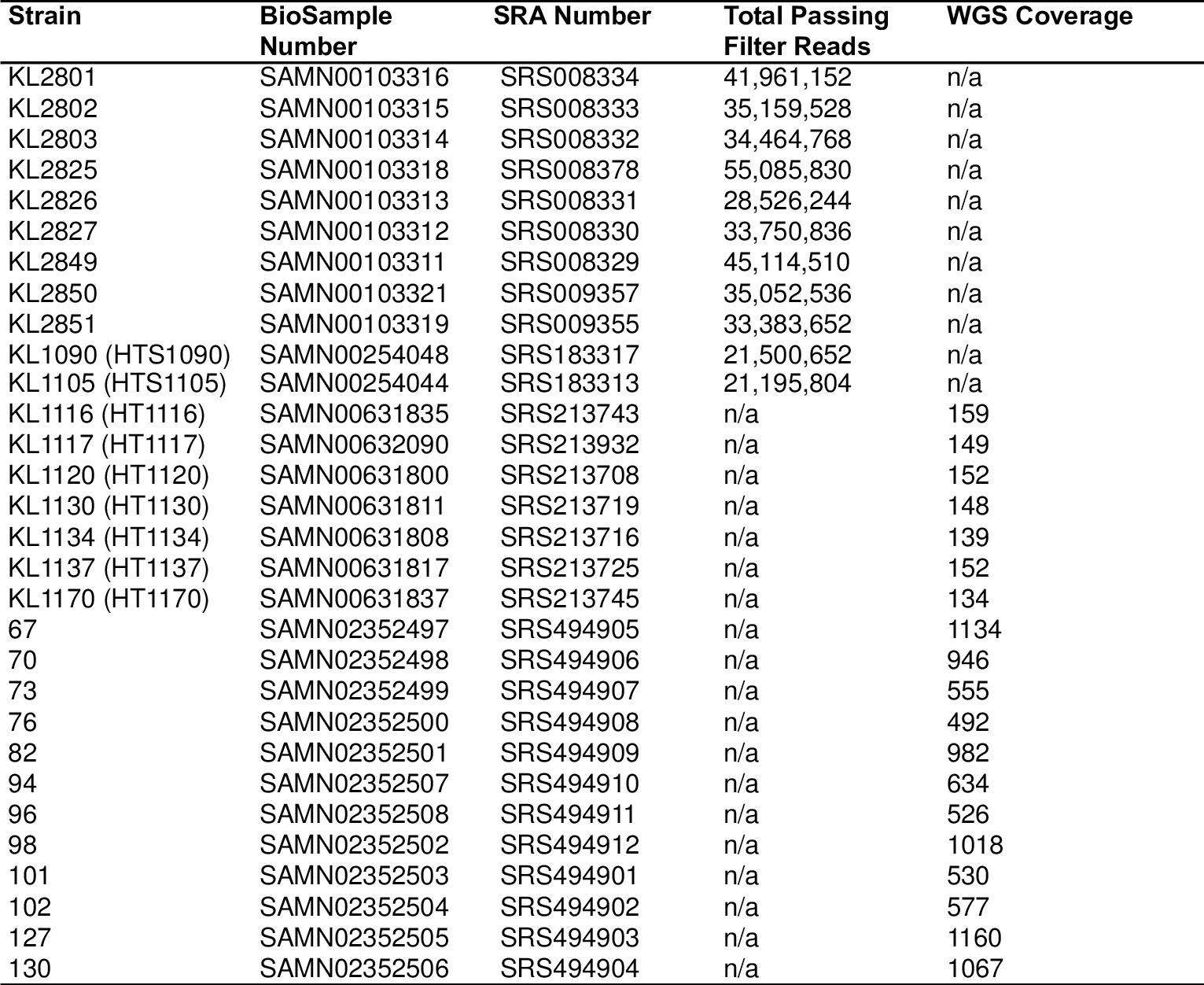

Supplement: S2 Table — (TIF) [file pone.0155127.s007.tif]

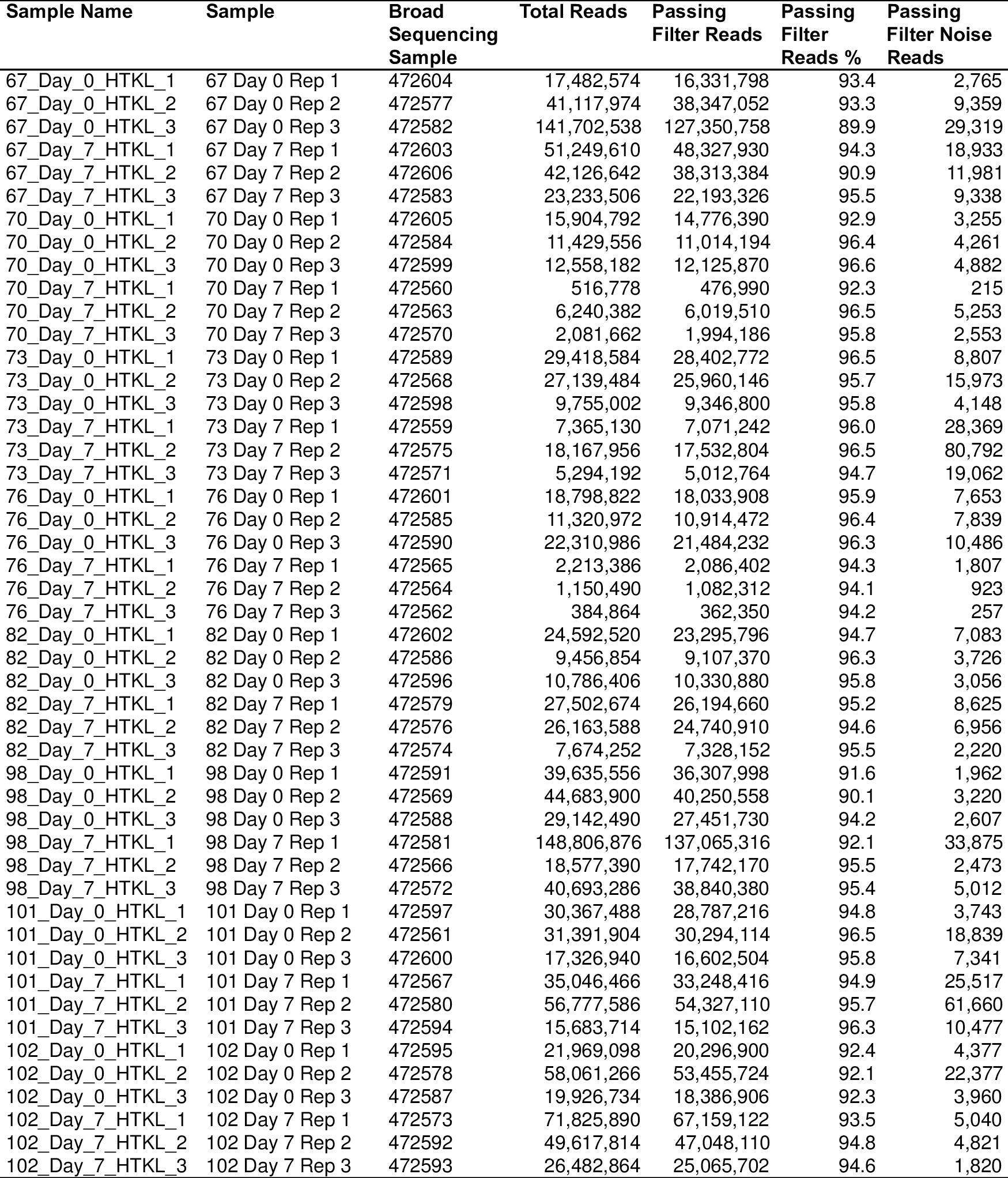

Supplement: S3 Table — (TIF) [file pone.0155127.s008.tif]

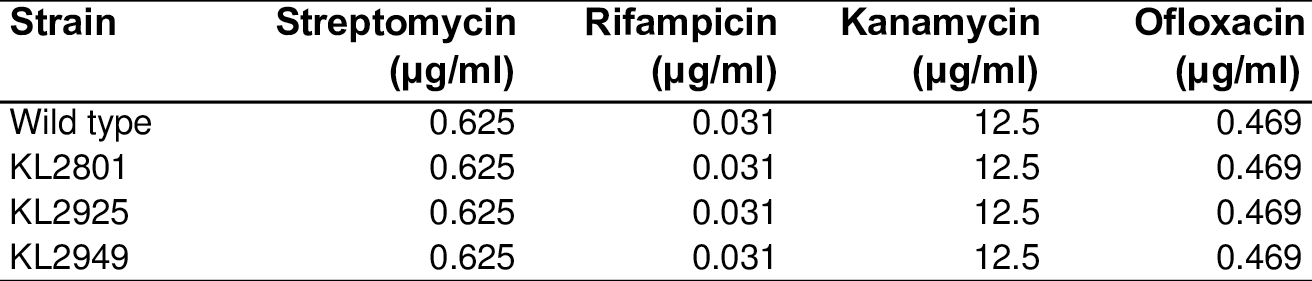

Supplement: S4 Table — (TIF) [file pone.0155127.s009.tif]

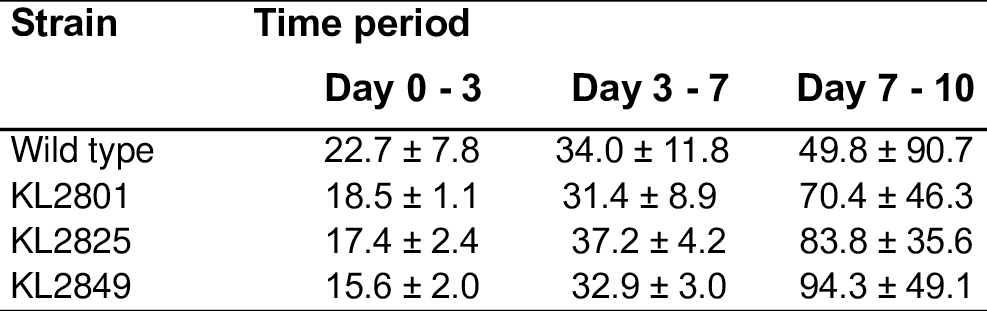

Supplement: S5 Table — (TIF) [file pone.0155127.s010.tif]

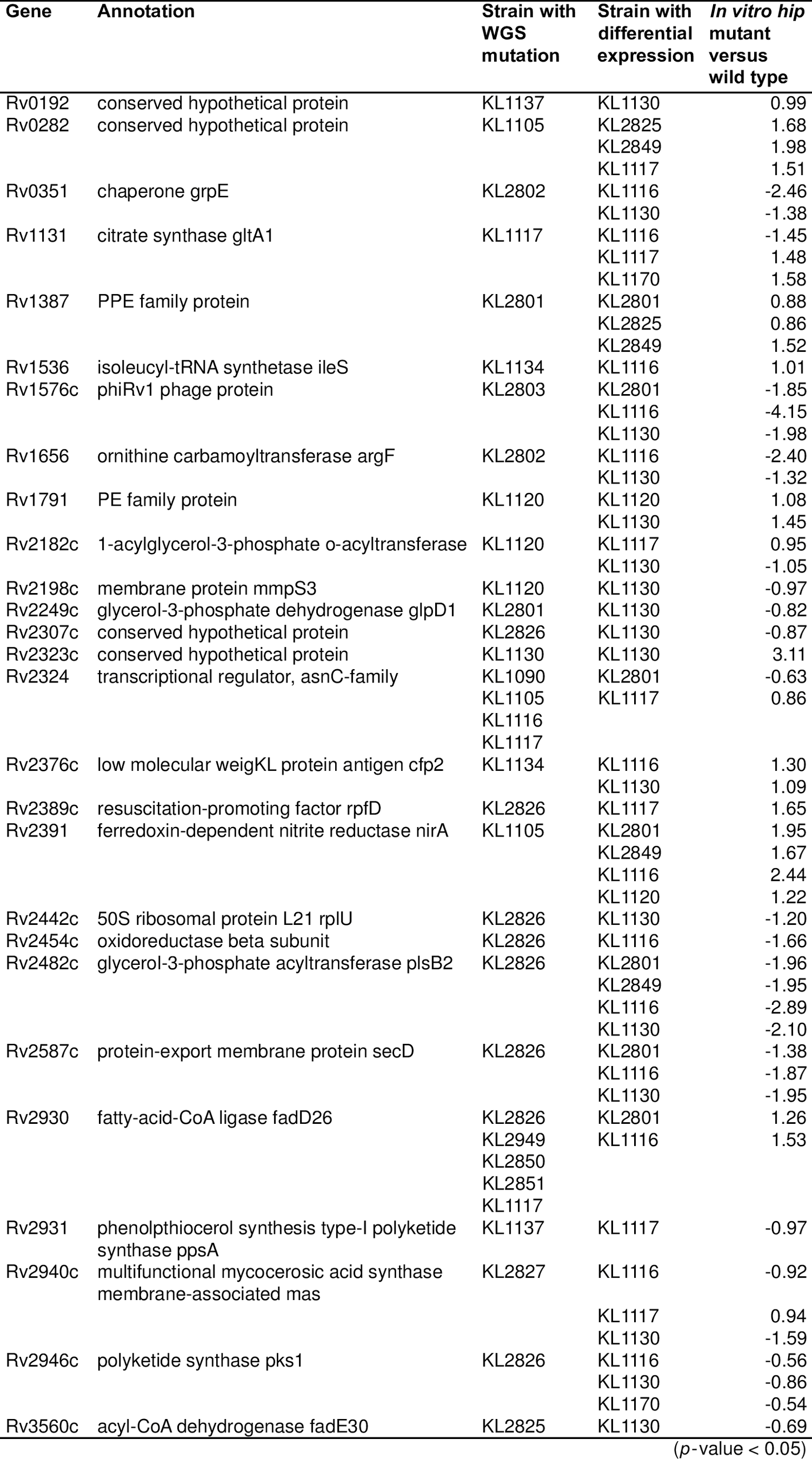

Supplement: S6 Table — (TIF) [file pone.0155127.s011.tif]

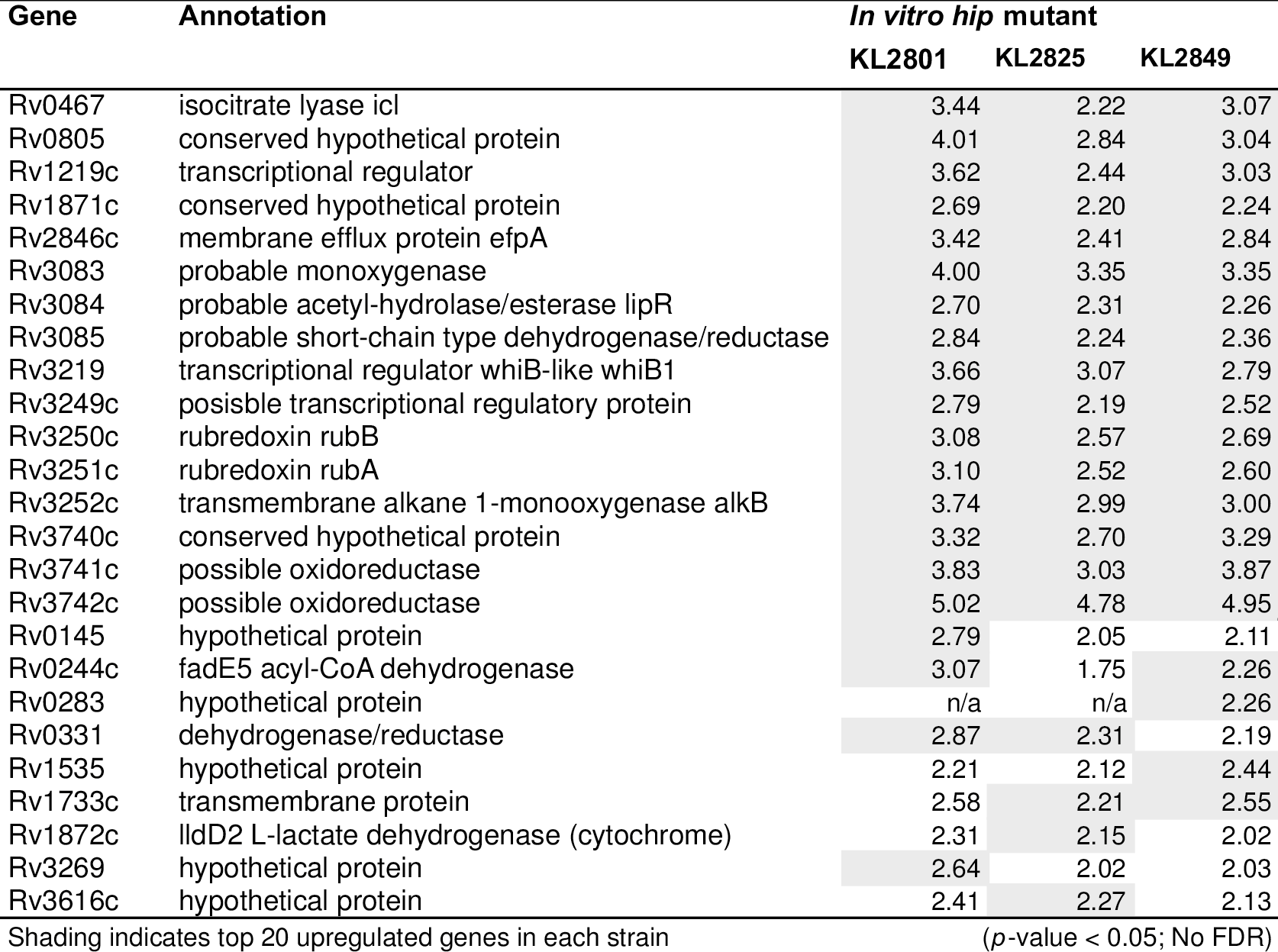

Supplement: S7 Table — (TIF) [file pone.0155127.s012.tif]

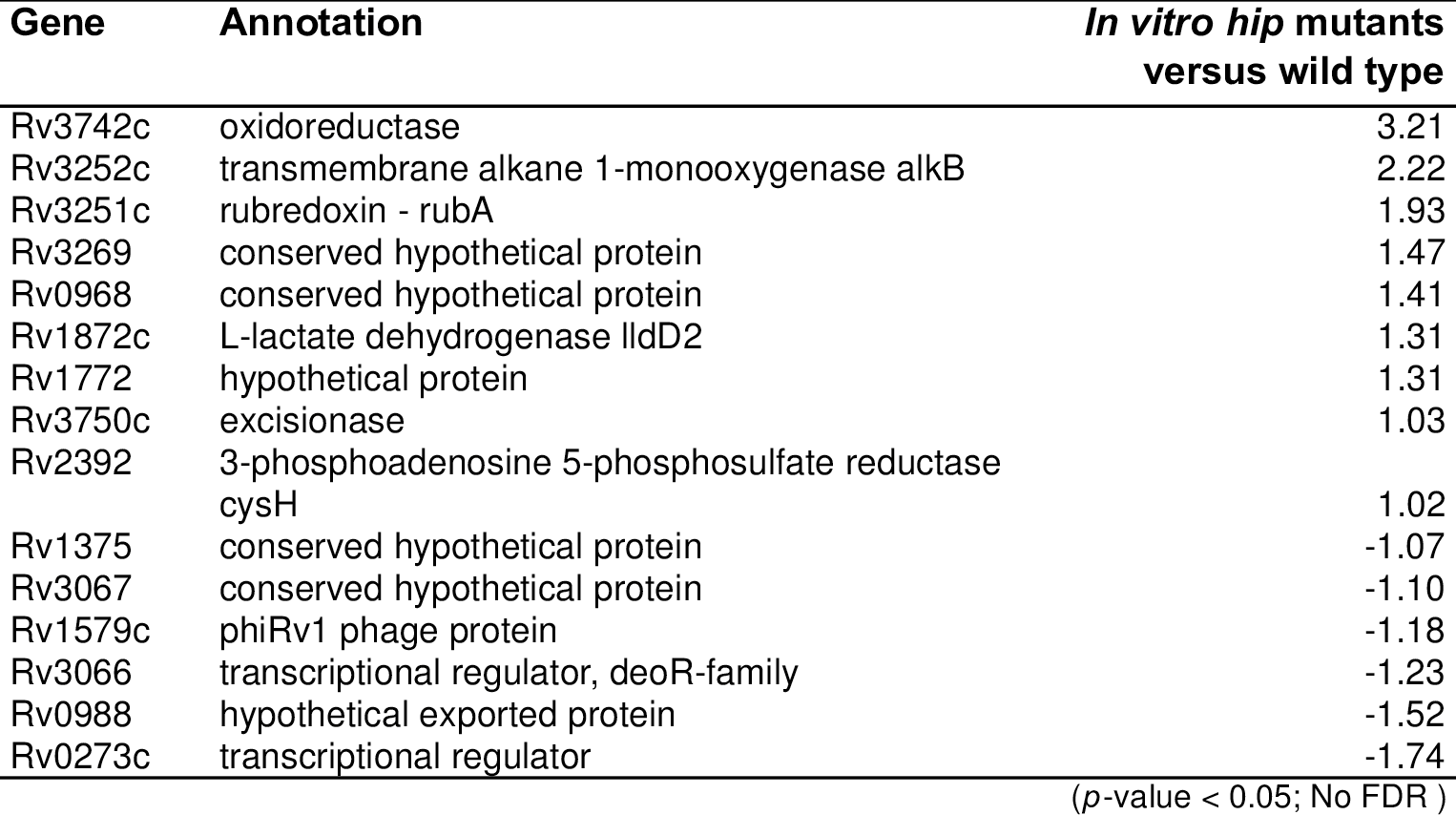

Supplement: S8 Table — (TIF) [file pone.0155127.s013.tif]

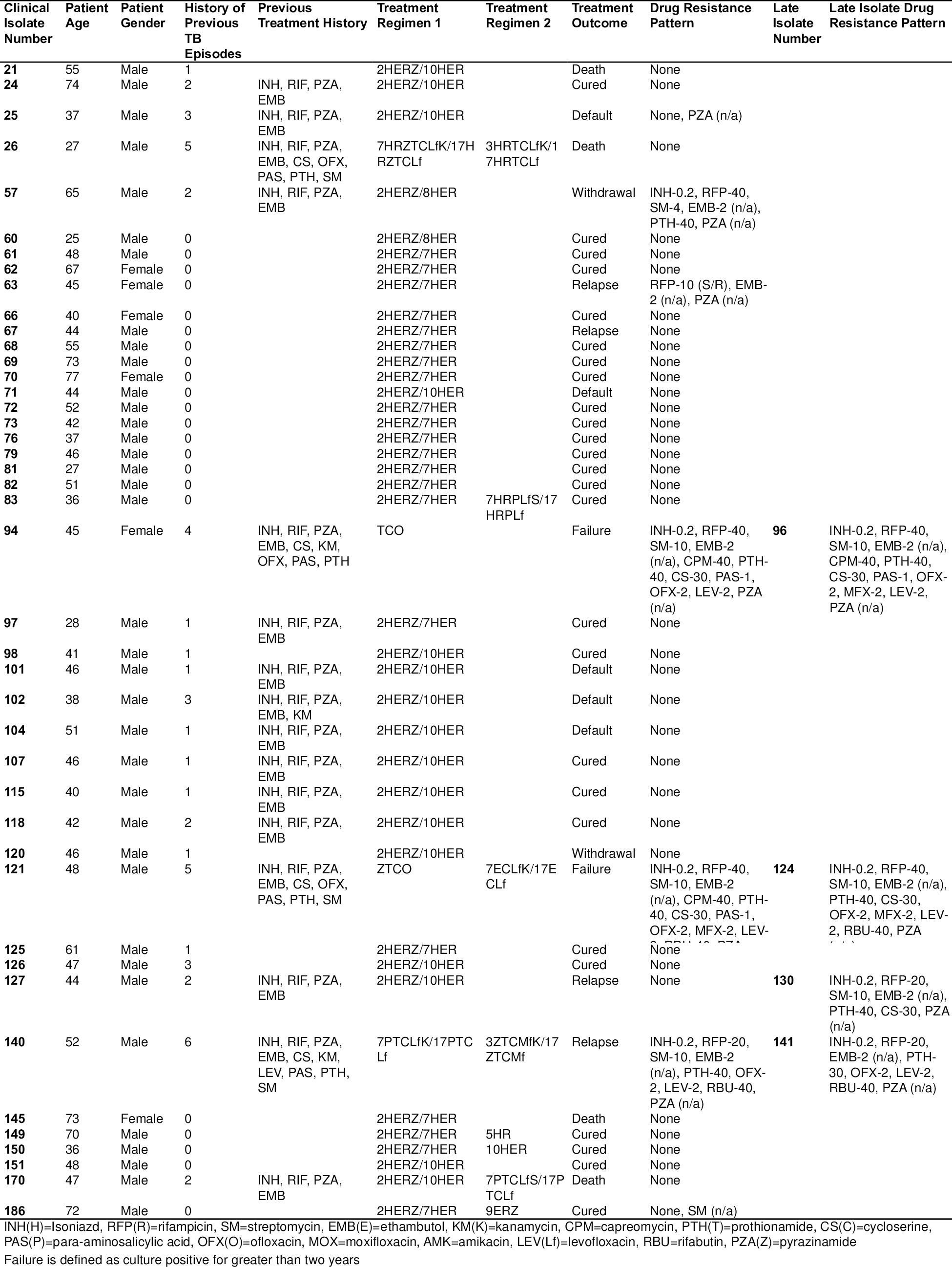

Supplement: S9 Table — (TIF) [file pone.0155127.s014.tif]

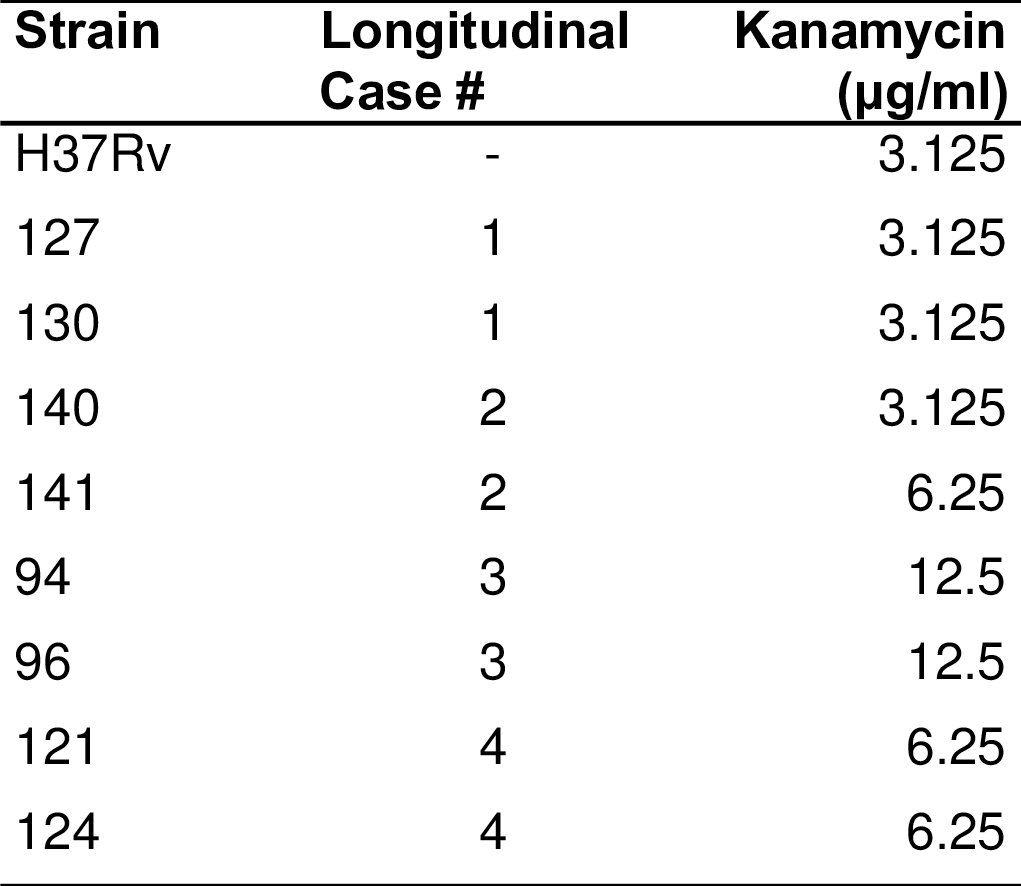

Supplement: S10 Table — (TIF) [file pone.0155127.s015.tif]

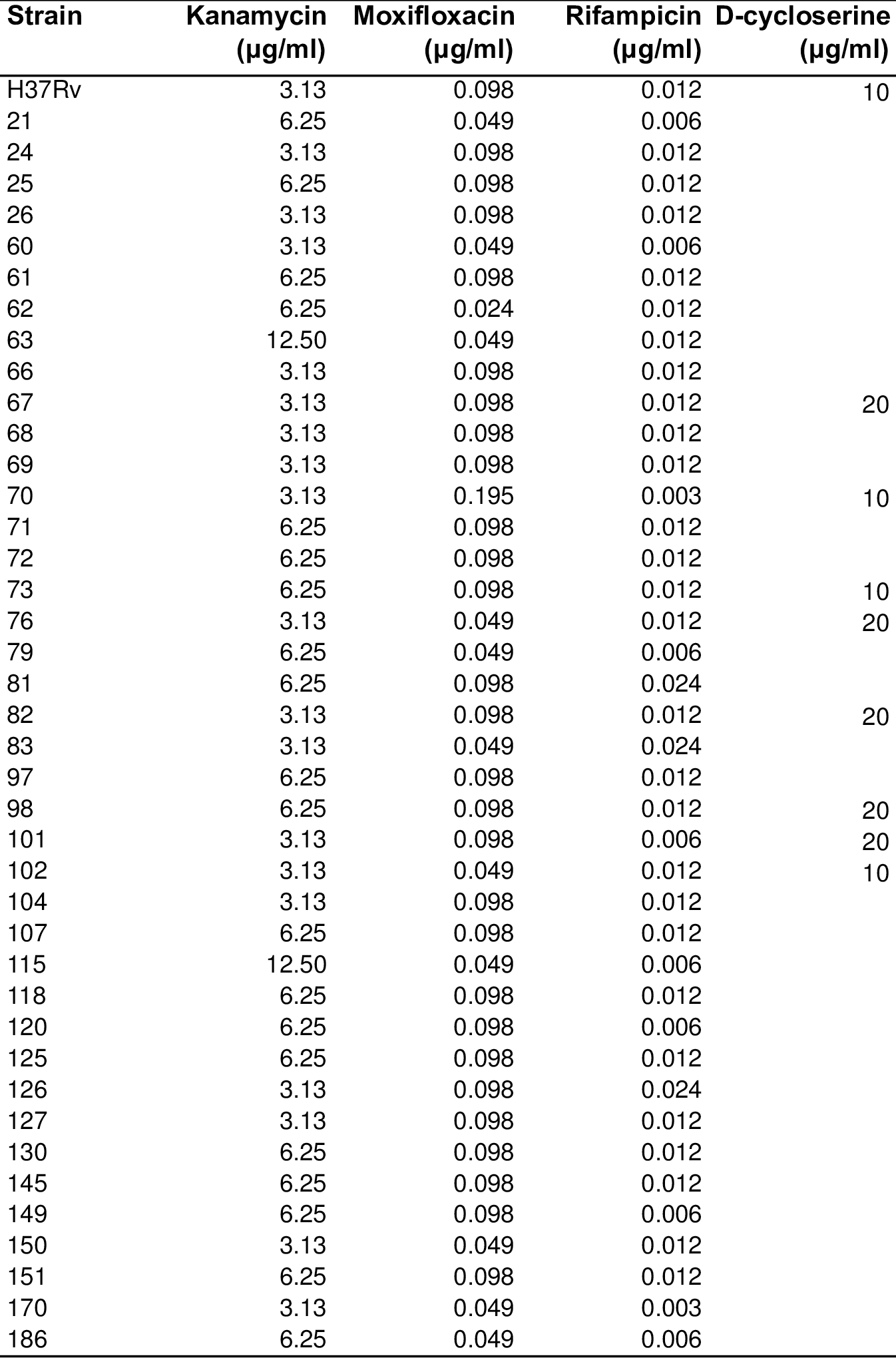

Supplement: S11 Table — (TIF) [file pone.0155127.s016.tif]

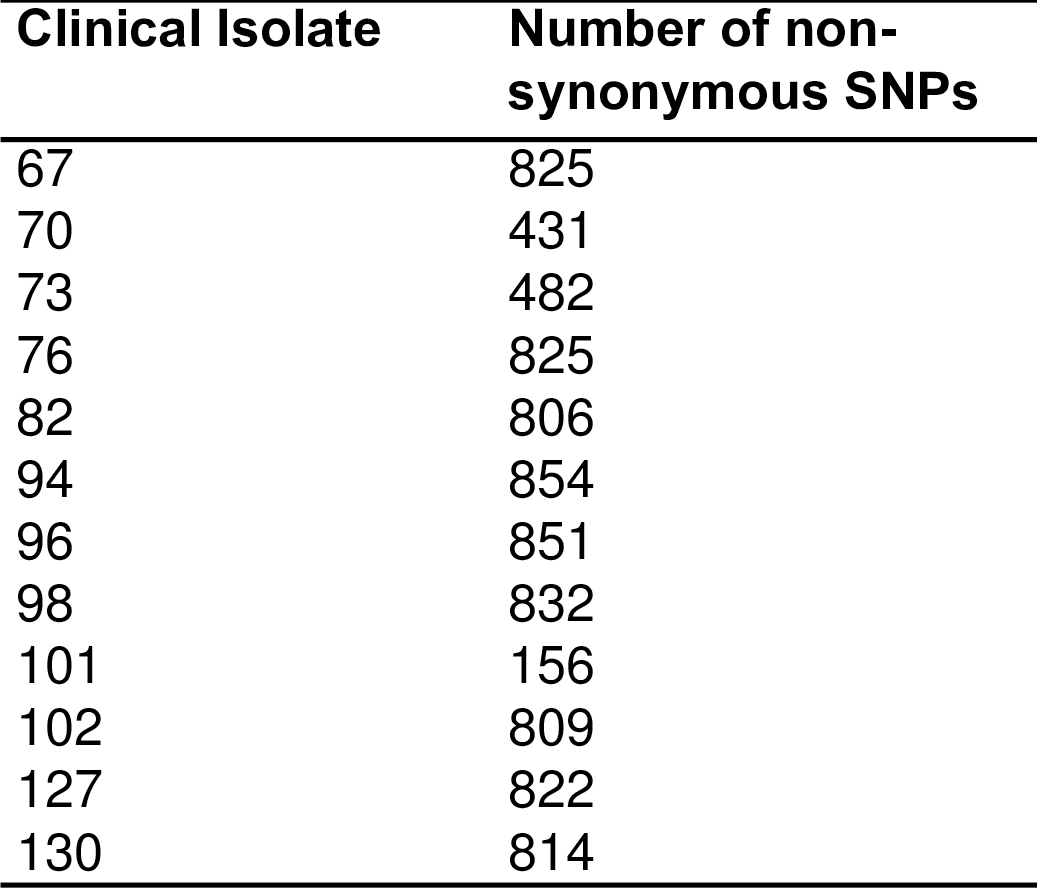

Supplement: S12 Table — (TIF) [file pone.0155127.s017.tif]

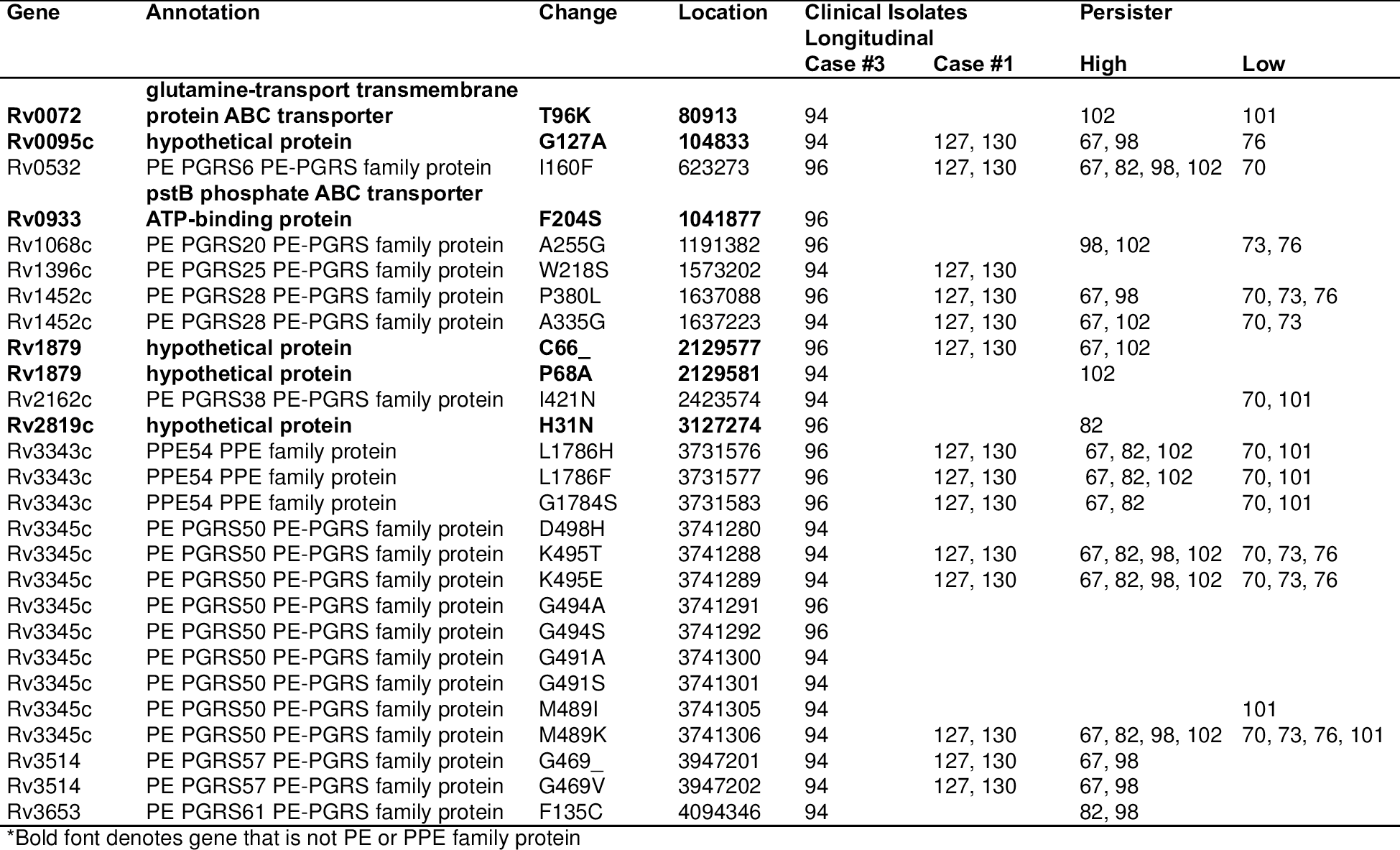

Supplement: S13 Table — (TIF) [file pone.0155127.s018.tif]

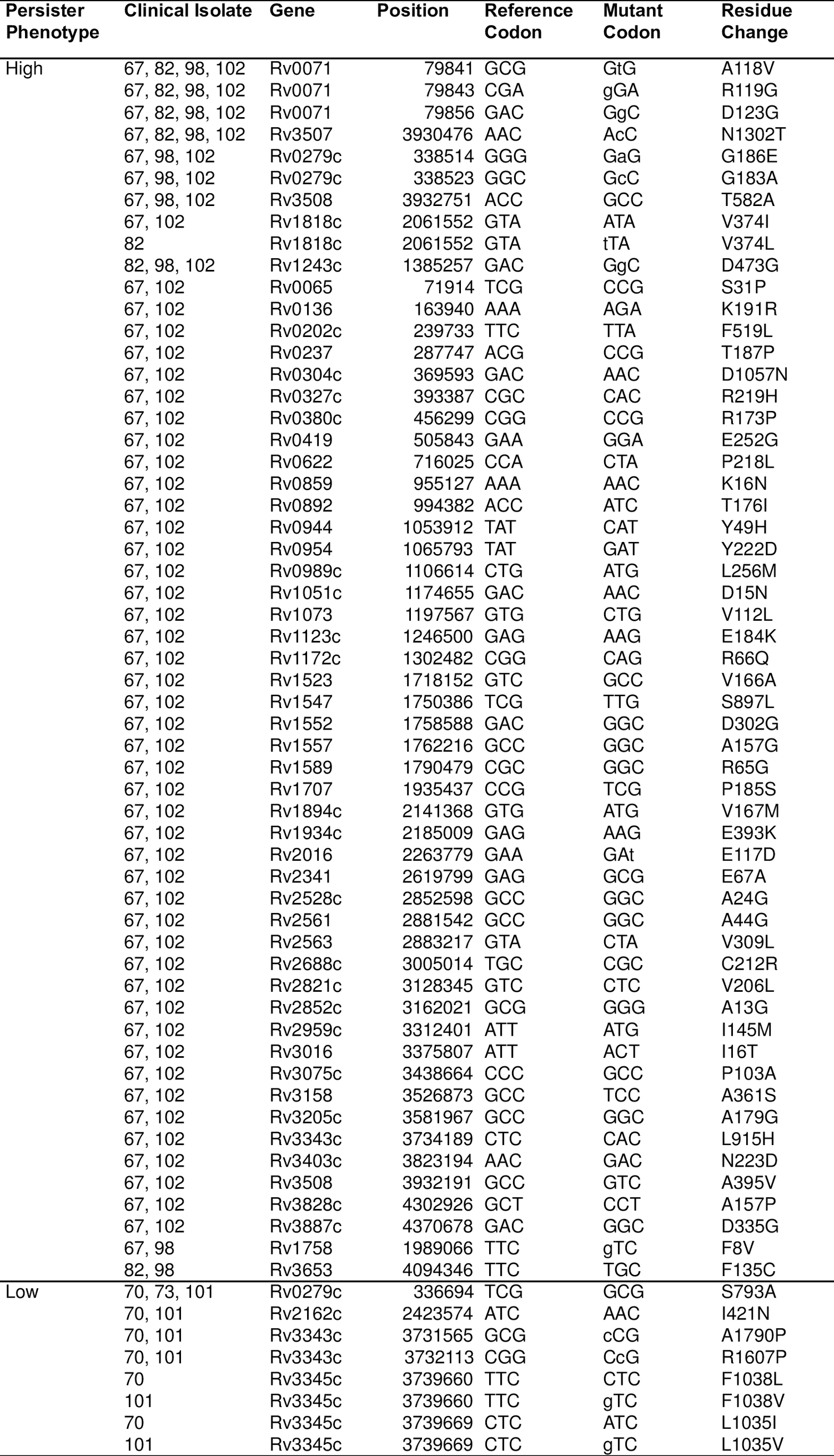

Supplement: S14 Table — (TIF) [file pone.0155127.s019.tif]

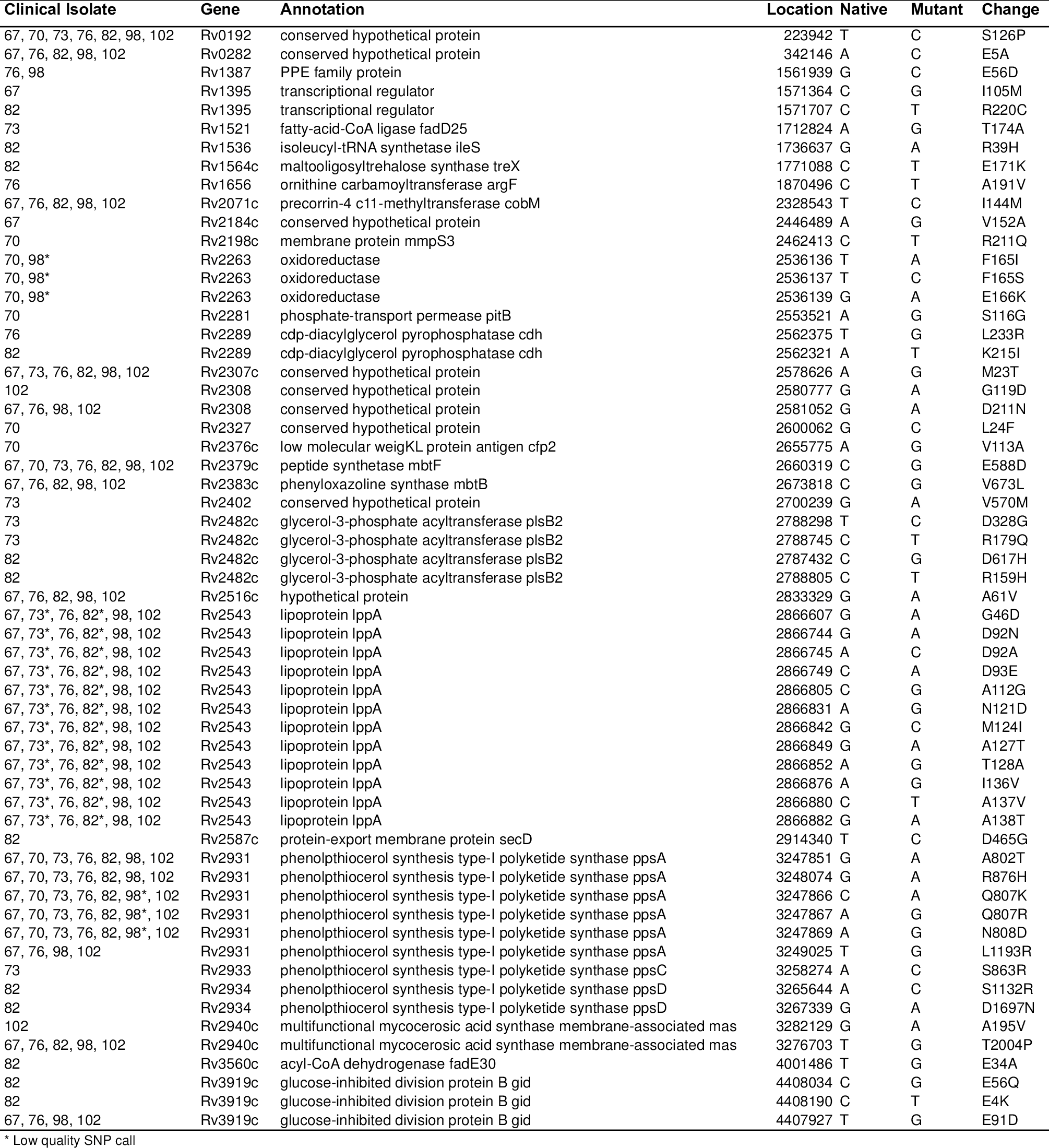

Supplement: S15 Table — (TIF) [file pone.0155127.s020.tif]

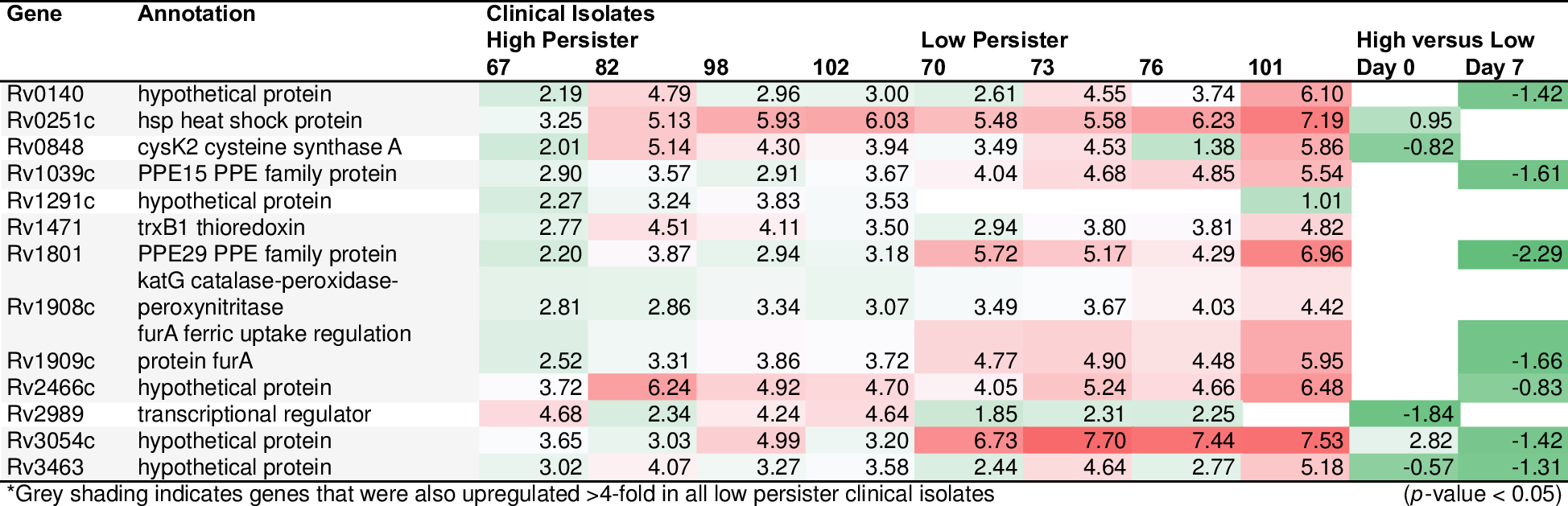

Supplement: S16 Table — (TIF) [file pone.0155127.s021.tif]

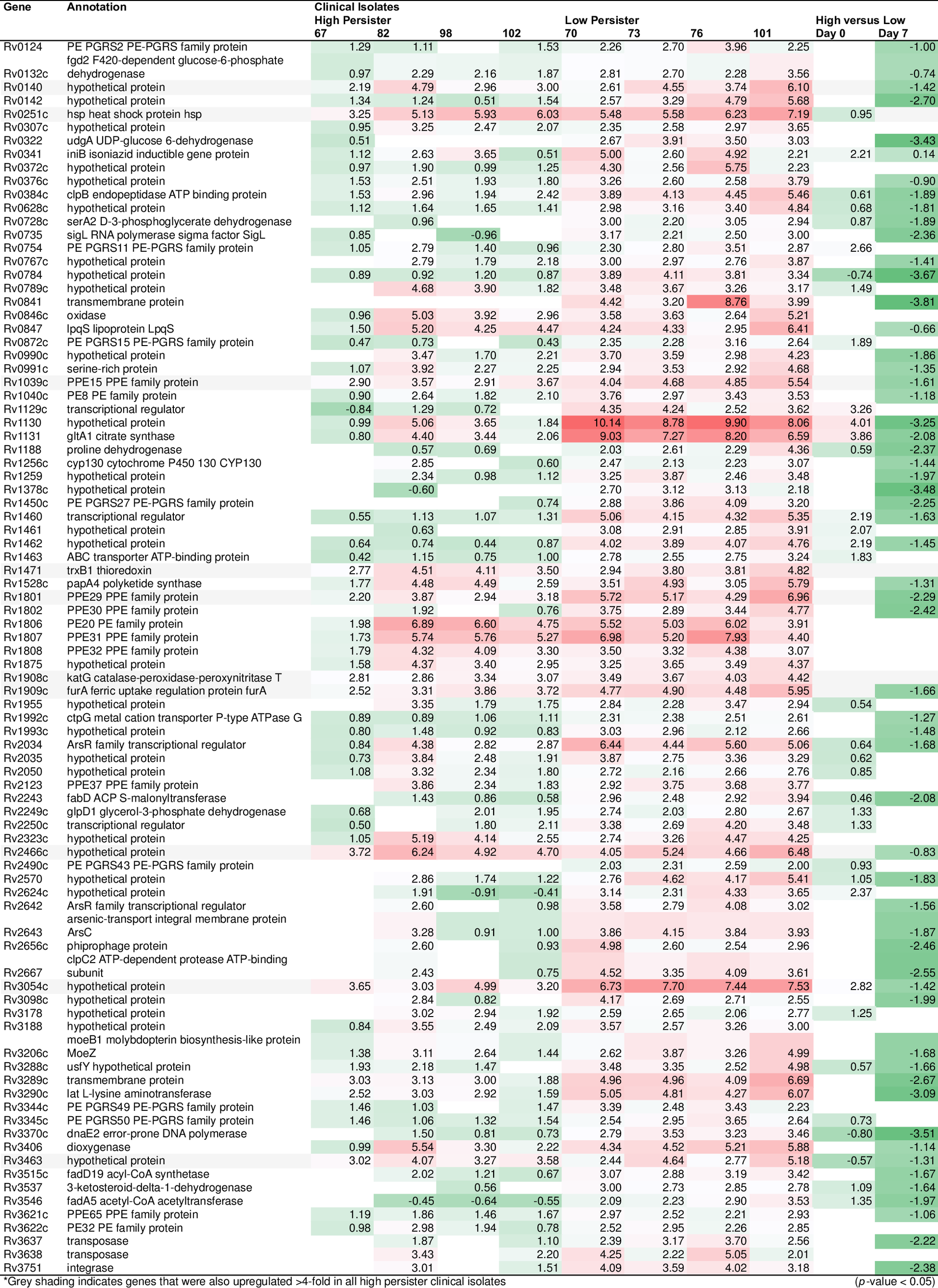

Supplement: S17 Table — (TIF) [file pone.0155127.s022.tif]

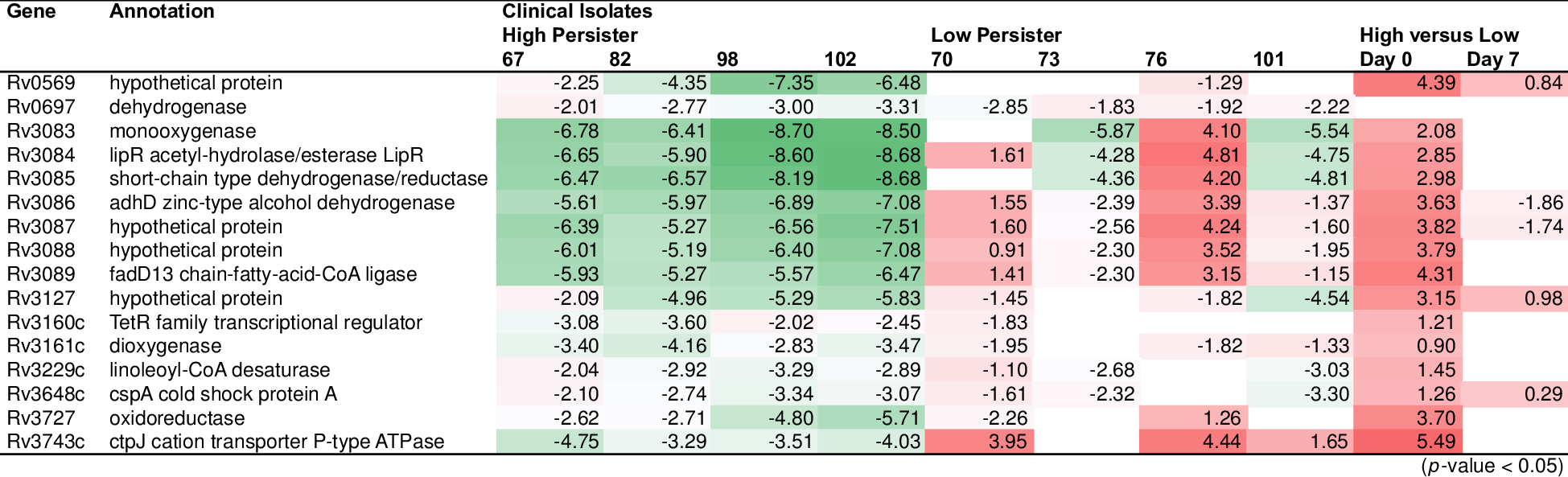

Supplement: S18 Table — (TIF) [file pone.0155127.s023.tif]

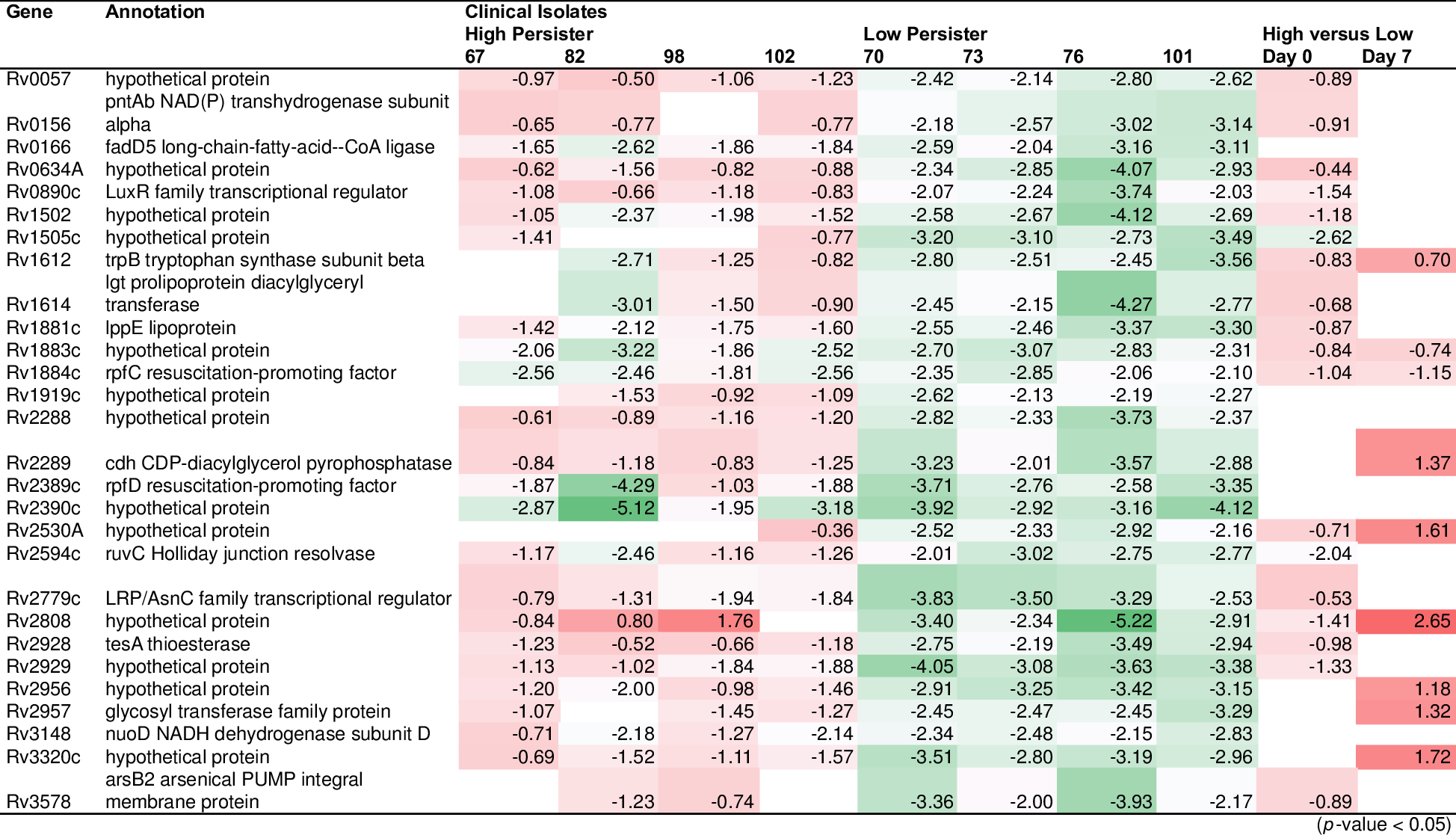

Supplement: S19 Table — (TIF) [file pone.0155127.s024.tif]

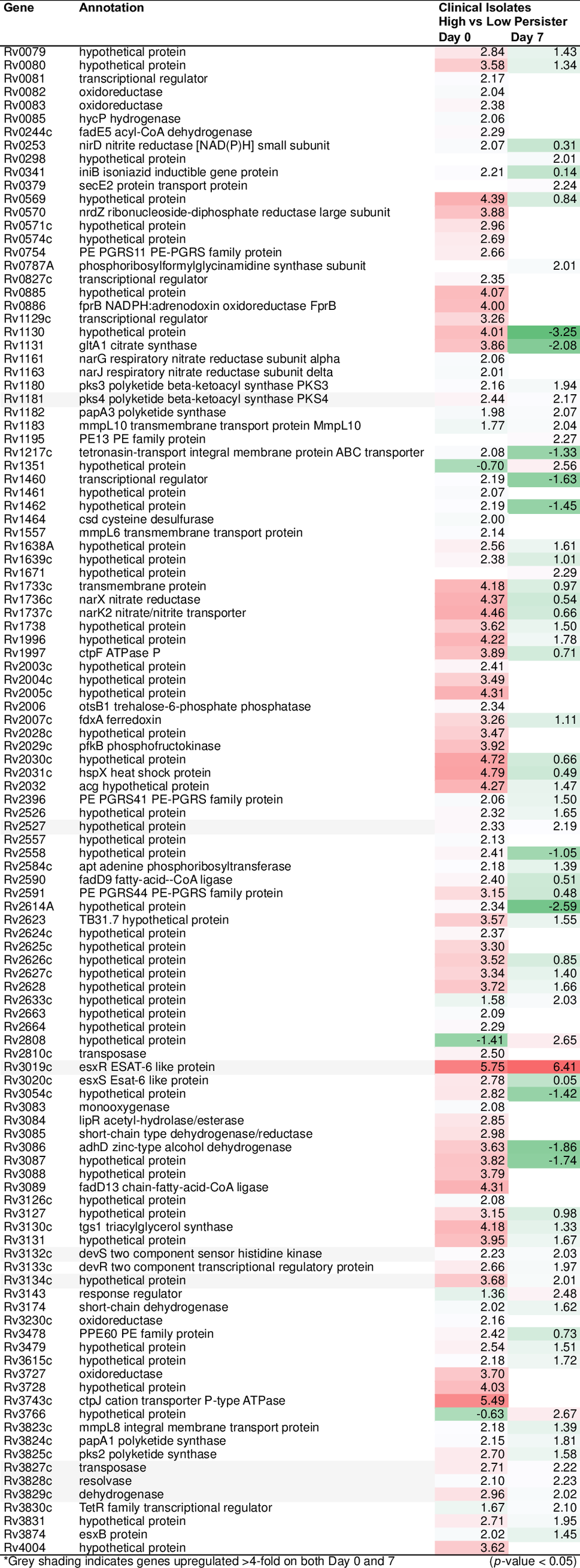

Supplement: S20 Table — (TIF) [file pone.0155127.s025.tif]

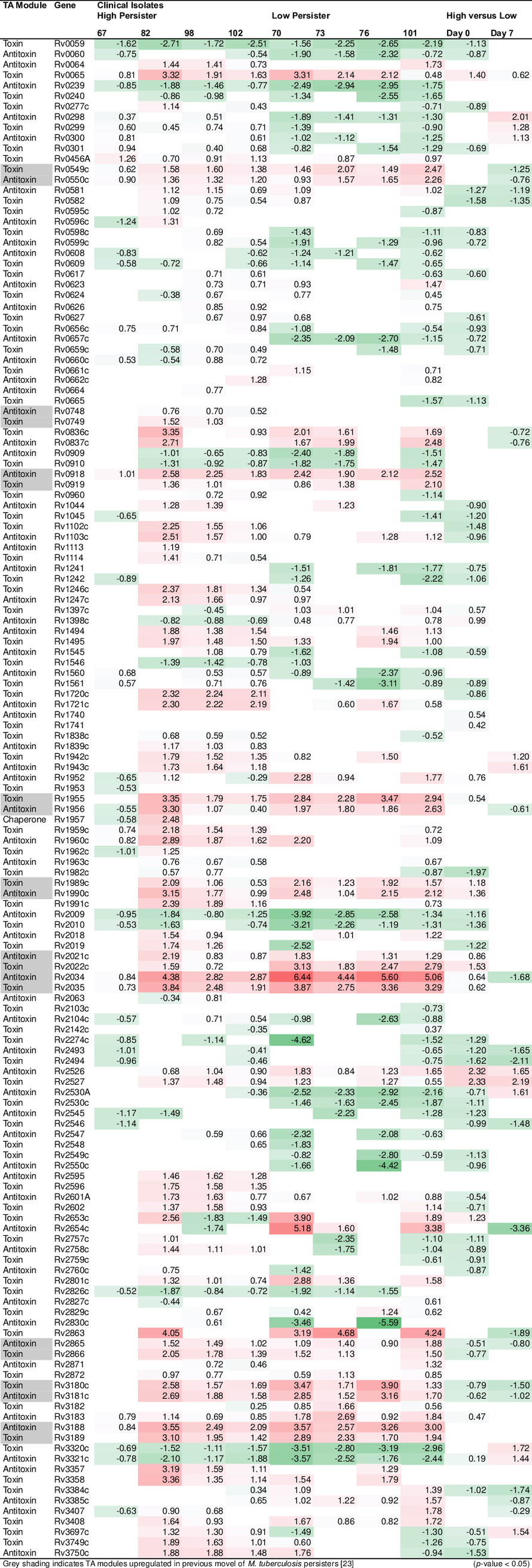

Supplement: S21 Table — (TIF) [file pone.0155127.s026.tif]

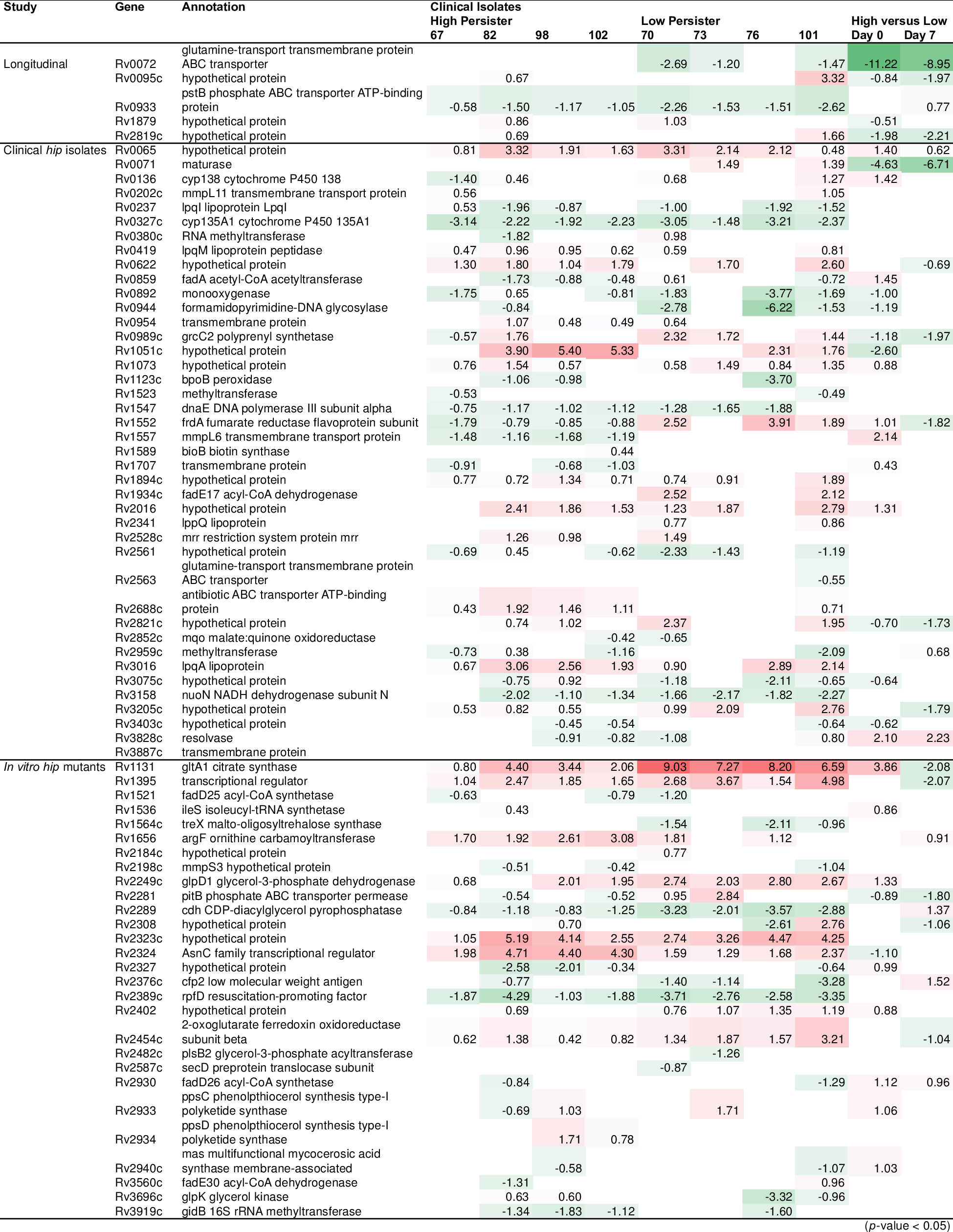

Supplement: S22 Table — (TIF) [file pone.0155127.s027.tif]

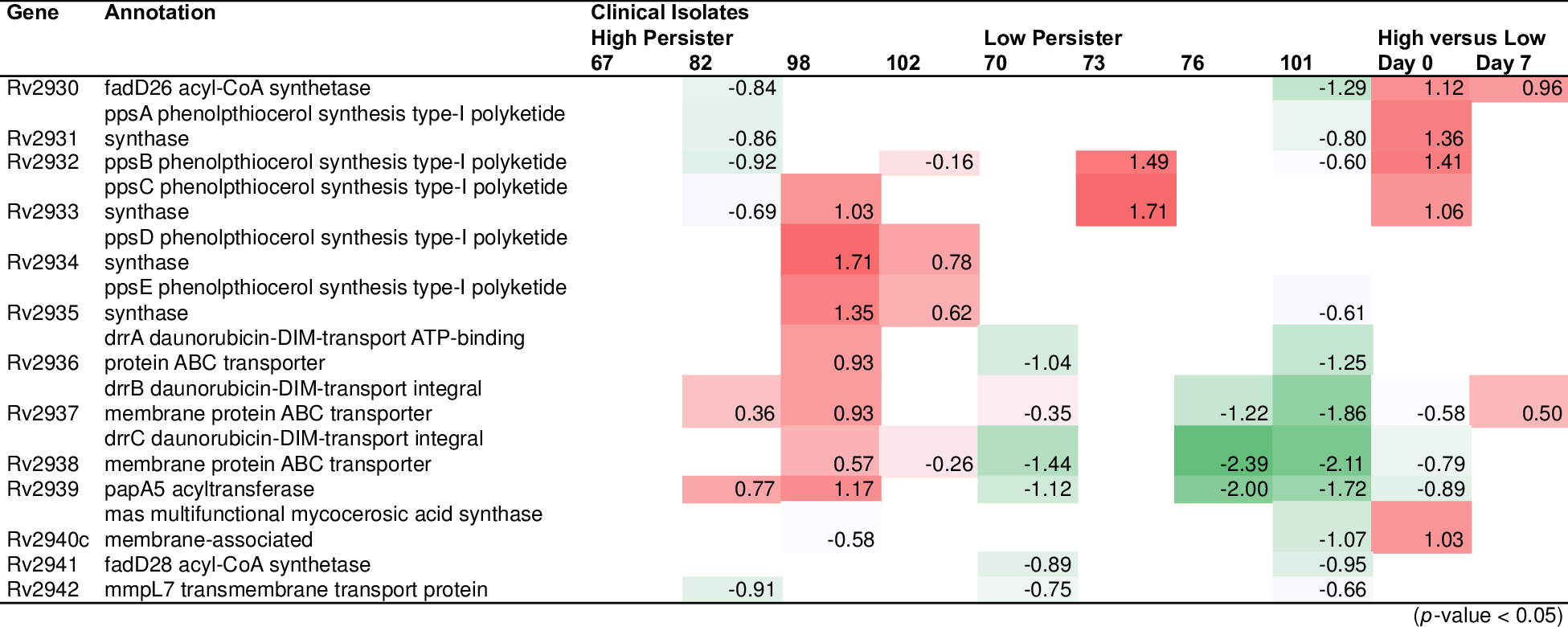

Supplement: S23 Table — (TIF) [file pone.0155127.s028.tif]

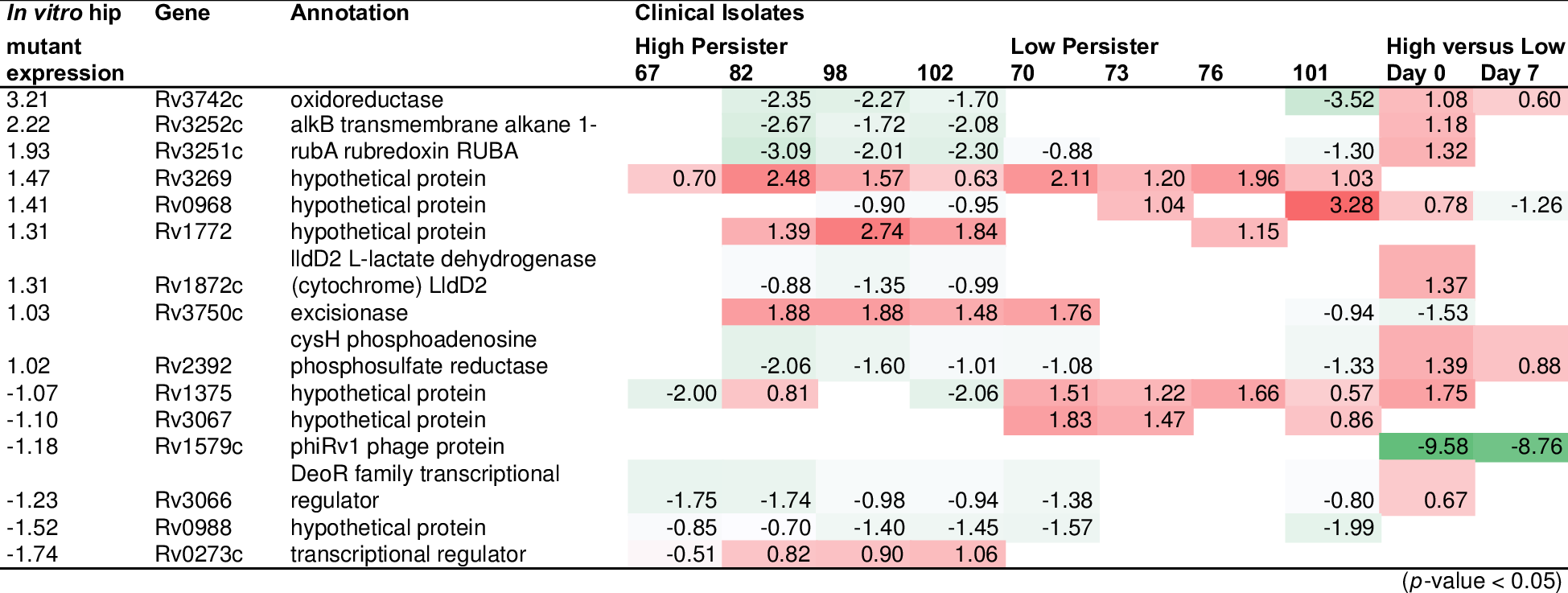

Supplement: S24 Table — (TIF) [file pone.0155127.s029.tif]

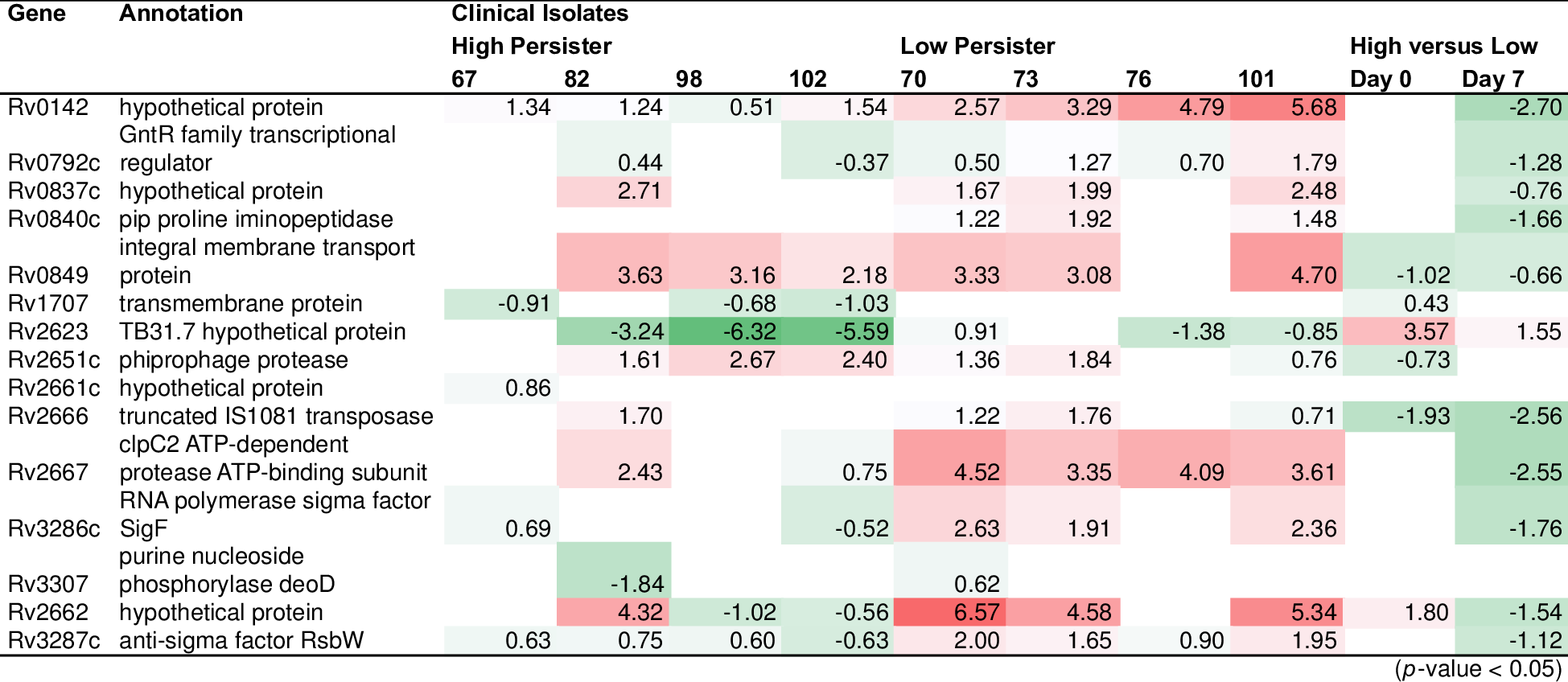

Supplement: S25 Table — (TIF) [file pone.0155127.s030.tif]
